# Conventional heart failure therapy in cardiac ATTR amyloidosis

**DOI:** 10.1093/eurheartj/ehad347

**Published:** 2023-05-22

**Authors:** Adam Ioannou, Paolo Massa, Rishi K Patel, Yousuf Razvi, Aldostefano Porcari, Muhammad U Rauf, Anita Jiang, Giacomo Cabras, Stefano Filisetti, Roos E Bolhuis, Francesco Bandera, Lucia Venneri, Ana Martinez-Naharro, Steven Law, Tushar Kotecha, Ruta Virsinskaite, Daniel S Knight, Michele Emdin, Aviva Petrie, Helen Lachmann, Ashutosh Wechelakar, Mark Petrie, Alun Hughes, Nick Freemantle, Philip N Hawkins, Carol Whelan, John J V McMurray, Julian D Gillmore, Marianna Fontana

**Affiliations:** National Amyloidosis Centre, University College London, Royal Free Campus, Rowland Hill Street, London NW3 2PF, UK; National Amyloidosis Centre, University College London, Royal Free Campus, Rowland Hill Street, London NW3 2PF, UK; National Amyloidosis Centre, University College London, Royal Free Campus, Rowland Hill Street, London NW3 2PF, UK; National Amyloidosis Centre, University College London, Royal Free Campus, Rowland Hill Street, London NW3 2PF, UK; National Amyloidosis Centre, University College London, Royal Free Campus, Rowland Hill Street, London NW3 2PF, UK; Center for Diagnosis and Treatment of Cardiomyopathies, Cardiovascular Department, Azienda Sanitaria Universitaria Giuliano-Isontina (ASUGI), University of Trieste, Via Giacomo Puccini, Trieste 34100, Italy; National Amyloidosis Centre, University College London, Royal Free Campus, Rowland Hill Street, London NW3 2PF, UK; National Amyloidosis Centre, University College London, Royal Free Campus, Rowland Hill Street, London NW3 2PF, UK; National Amyloidosis Centre, University College London, Royal Free Campus, Rowland Hill Street, London NW3 2PF, UK; Cardiology University Department, IRCCS Policlinico San Donato, Piazza Edmondo Malan, Milan 20097, Italy; National Amyloidosis Centre, University College London, Royal Free Campus, Rowland Hill Street, London NW3 2PF, UK; Cardiology University Department, IRCCS Policlinico San Donato, Piazza Edmondo Malan, Milan 20097, Italy; National Amyloidosis Centre, University College London, Royal Free Campus, Rowland Hill Street, London NW3 2PF, UK; National Amyloidosis Centre, University College London, Royal Free Campus, Rowland Hill Street, London NW3 2PF, UK; National Amyloidosis Centre, University College London, Royal Free Campus, Rowland Hill Street, London NW3 2PF, UK; National Amyloidosis Centre, University College London, Royal Free Campus, Rowland Hill Street, London NW3 2PF, UK; National Amyloidosis Centre, University College London, Royal Free Campus, Rowland Hill Street, London NW3 2PF, UK; National Amyloidosis Centre, University College London, Royal Free Campus, Rowland Hill Street, London NW3 2PF, UK; Health Science Interdisciplinary Center, Scuola Superiore Sant’Anna, Via Giuseppe Moruzzi, Pisa 56127, Italy; Cardiovascular Department, Fondazione Toscana Gabriele Monasterio, Via Giuseppe Moruzzi, Pisa 56124, Italy; Biostatistics Unit, University College London Eastman Dental Institute, 256 Grays Inn Road, London WC1X 8LD, UK; National Amyloidosis Centre, University College London, Royal Free Campus, Rowland Hill Street, London NW3 2PF, UK; National Amyloidosis Centre, University College London, Royal Free Campus, Rowland Hill Street, London NW3 2PF, UK; BHF Cardiovascular Research Centre, University of Glasgow, 126 University Pl, Glasgow G12 8TA, UK; Institute of Cardiovascular Science, University College London, 1–19 Torrington Place, London WC1E 7HB, UK; University College London, London, UK; National Amyloidosis Centre, University College London, Royal Free Campus, Rowland Hill Street, London NW3 2PF, UK; National Amyloidosis Centre, University College London, Royal Free Campus, Rowland Hill Street, London NW3 2PF, UK; BHF Cardiovascular Research Centre, University of Glasgow, 126 University Pl, Glasgow G12 8TA, UK; National Amyloidosis Centre, University College London, Royal Free Campus, Rowland Hill Street, London NW3 2PF, UK; National Amyloidosis Centre, University College London, Royal Free Campus, Rowland Hill Street, London NW3 2PF, UK

**Keywords:** Cardiac ATTR amyloidosis, Heart failure, Heart failure medications, Beta-blockers, Mineralocorticoid receptor antagonists

## Abstract

**Aims:**

The aims of this study were to assess prescription patterns, dosages, discontinuation rates, and association with prognosis of conventional heart failure medications in patients with transthyretin cardiac amyloidosis (ATTR-CA).

**Methods and results:**

A retrospective analysis of all consecutive patients diagnosed with ATTR-CA at the National Amyloidosis Centre between 2000 and 2022 identified 2371 patients with ATTR-CA. Prescription of heart failure medications was greater among patients with a more severe cardiac phenotype, comprising beta-blockers in 55.4%, angiotensin-converting enzyme inhibitors (ACEis)/angiotensin II receptor blockers (ARBs) in 57.4%, and mineralocorticoid receptor antagonists (MRAs) in 39.0% of cases. During a median follow-up of 27.8 months (interquartile range 10.6–51.3), 21.7% had beta-blockers discontinued, and 32.9% had ACEi/ARBs discontinued. In contrast, only 7.5% had MRAs discontinued. A propensity score-matched analysis demonstrated that treatment with MRAs was independently associated with a reduced risk of mortality in the overall population [hazard ratio (HR) 0.77 (95% confidence interval (CI) 0.66–0.89), *P* < .001] and in a pre-specified subgroup of patients with a left ventricular ejection fraction (LVEF) >40% [HR 0.75 (95% CI 0.63–0.90), *P* = .002]; and treatment with low-dose beta-blockers was independently associated with a reduced risk of mortality in a pre-specified subgroup of patients with a LVEF ≤40% [HR 0.61 (95% CI 0.45–0.83), *P* = .002]. No convincing differences were found for treatment with ACEi/ARBs.

**Conclusion:**

Conventional heart failure medications are currently not widely prescribed in ATTR-CA, and those that received medication had more severe cardiac disease. Beta-blockers and ACEi/ARBs were often discontinued, but low-dose beta-blockers were associated with reduced risk of mortality in patients with a LVEF ≤40%. In contrast, MRAs were rarely discontinued and were associated with reduced risk of mortality in the overall population; but these findings require confirmation in prospective randomized controlled trials.

## Structured Graphical Abstract

**Structured Graphical Abstract:**
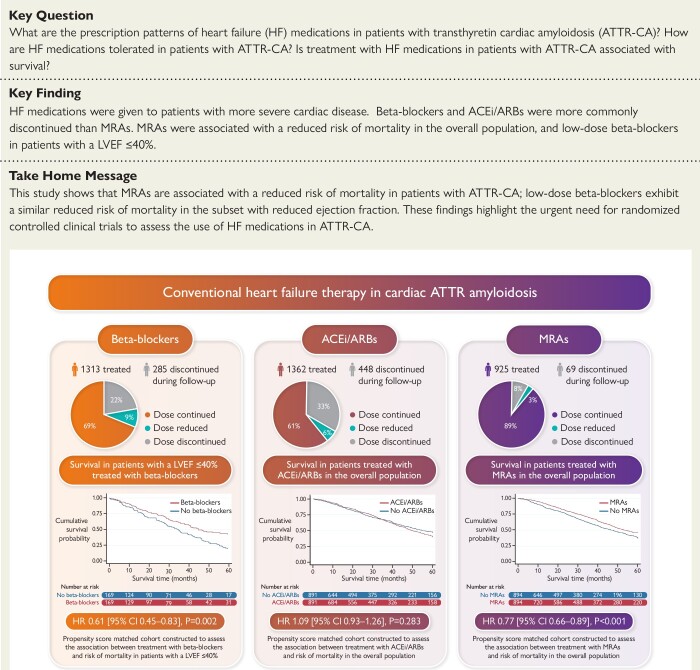
Discontinuation rates of heart failure medications in patients with cardiac ATTR amyloidosis. Kaplan–Meier curves comparing survival in patients treated with heart failure medications to propensity score-matched patients not treated with heart failure medications, followed by a Cox proportional hazards regression analysis. ACEi, angiotensin-converting enzyme inhibitor; ARBs, angiotensin II receptor blockers; MRAs, mineralocorticoid receptor antagonists; LVEF, left ventricular ejection fraction; HR, hazard ratio; CI, confidence interval.

**See the editorial comment for this article ‘Neurohormonal blockade in transthyretin amyloidosis: perhaps one size does not fit all?’, by R.K. Cheng and S.A.M. Cuddy, https://doi.org/10.1093/eurheartj/ehad357.**

## Introduction

Transthyretin cardiac amyloidosis (ATTR-CA) causes progressive, fatal, heart failure (HF), due to misfolding of transthyretin (TTR), forming insoluble amyloid fibrils, which are deposited within the myocardial extracellular space.^1,2^ Until recently, ATTR-CA was considered a rare, untreatable disease. However, improvements in diagnostics coupled with emerging high-cost therapies are challenging these long-held beliefs. The ATTR-CA is far more common than previously suspected, and there is a potential for successful therapeutic intervention.^3^

The only drug proved to be associated with prognostic benefit in ATTR-CA is tafamidis, which is a highly specific drug that targets the circulating TTR protein and stabilizes the TTR tetramer to prevent dissociation into amyloidogenic monomers that deposit in the myocardium, causing an infiltrative and restrictive cardiomyopathy. Tafamidis was shown in a phase 3 placebo-controlled trial (ATTR-ACT) to reduce the combined primary endpoint of cardiovascular hospitalizations and mortality.^4^ However, unfortunately, the high cost associated with tafamidis has resulted in restricted use, and tafamidis has not been approved for the treatment of ATTR-CA in many countries.^5^

At present, it is unknown whether conventional HF medications that have substantial benefits in patients with HF of other aetiologies may also benefit those with ATTR-CA, as patients with known ATTR-CA have been excluded from previous HF trials.^6–13^ Hence, the value of conventional HF medications in patients with ATTR-CA is still debated. Small-scale studies have yielded contrasting results, with some suggesting that low doses of conventional HF medications are well tolerated,^14,15^ while others reported that not only are these medications poorly tolerated, but they may result in worse outcomes.^16,17^ The lack of large-scale clinical trials has resulted in a significant knowledge gap, although a position statement from the European Society of Cardiology (ESC) working group on myocardial and pericardial diseases regarding HF medications in ATTR-CA recommends stopping beta-blockers, and avoiding angiotensin-converting enzyme inhibitors (ACEis) and angiotensin II receptor blockers (ARBs), and are silent about mineralocorticoid receptor antagonists (MRAs).^18^

The aims of this study were to: (i) assess the prescription pattern of conventional HF medications in patients with ATTR-CA; (ii) assess the dosages and discontinuation rates of HF medications in patients with ATTR-CA; and (iii) assess the association between treatment with HF medications and survival in patients with ATTR-CA.

## Methods

Consecutive patients in whom a diagnosis of ATTR-CA was confirmed at the National Amyloidosis Centre (NAC), between January 2000 and September 2022, were included. Patients with evidence of ATTR-polyneuropathy were excluded, as many have autonomic neuropathy and are not treated with HF medications due to concomitant postural hypotension.

Between 2000 and 2005 the diagnosis of ATTR-CA was established based on HF symptoms together with a characteristic CA echocardiogram and either direct endomyocardial biopsy proof of ATTR-amyloid or ATTR-amyloid in an extra-cardiac biopsy. From 2006 onwards cardiac magnetic resonance was added to the assessment if there was diagnostic doubt. From 2010 onwards, ^99m^Technetium labelled 3,3-diphosphono-1,2-propanodicarboxylic acid (^99m^Tc-DPD) scintigraphy was utilized, and diagnosis established based on ATTR-amyloid in an extra-cardiac biopsy with cardiac uptake on ^99m^Tc-DPD scintigraphy; or grade 2–3 cardiac uptake on ^99m^Tc-DPD scintigraphy in the absence of biochemical evidence of a plasma cell dyscrasia. All patients underwent genetic sequencing of the *TTR* gene and provided written consent for their data to be retrospectively analysed and published, in line with the Declaration of Helsinki and approval from the Royal Free Hospital ethics committee (REC 21/PR/0620).

All patients are enrolled into a protocolized follow-up program that consists of 6–12 monthly consultations. Data regarding whether HF medications were initiated, continued, or stopped, and medication dosages were all recorded. Medication classes were defined based on the ESC HF guidelines and comprised beta-blockers, ACEi/ARBs and MRAs. Target doses from the guidelines enabled comparisons by converting the daily dose to a percentage of the target dose. Medication classes were recorded regardless of whether the specific drug had been used in previous HF trials.^19^ Management decisions utilized a combined decision-making process involving local clinicians and the NAC team. Considering the knowledge gap, decisions concerning the initiation or discontinuation of HF medications were made following each clinical assessment on a case-by-case basis.

### Statistical analysis

Statistical analysis was performed using Stata (StataCorp. 2021. Stata Statistical Software: Release 17. College Station, TX: StataCorp LLC). All continuous variables were tested for normality (Shapiro–Wilk test) and presented as mean ± standard deviation if the distribution was normal or median [interquartile range (IQR)] otherwise, other than N-terminal pro-B-type natriuretic peptide (NT-proBNP) which was log-transformed for bivariate testing. The independent sample *t-*test was used to compare means if the data were normally distributed in each treatment group, or its non-parametric equivalent was used to compare the distributions of the two treatment groups. One-way analysis of variance if the data were normally distributed in each treatment group was used to compare means in more than two groups; or its non-parametric equivalent was used to compare the distributions of multiple groups. A significant result was followed by *post hoc* Bonferroni corrected pairwise comparisons to establish where differences lay. Categorical data are presented as absolute numbers and frequencies (%) and compared using the χ^2^ test.

All mortality data were obtained via the UK Office of National Statistics, which is the formal government registry for all deaths throughout the UK. The mortality endpoint was defined as time to death from date of diagnosis for all deceased patients and time to censor date (25 October 2022) from date of diagnosis among the remainder. Follow-up was restricted to ≤60 months, after which patients were censored due to the majority of events occurring in the first 60 months, and a low number of patients at risk after 60 months. To account for amyloid-specific disease-modifying therapy or clinical trials, patients were censored at their start date.

Survival was evaluated using Cox proportional hazards regression analysis, providing estimated hazard ratios (HRs) with 95% confidence intervals (CIs). The proportional hazards assumption was checked and confirmed using weighted Schoenfeld residuals. With regard to the survival analysis, patients were classed as being treated with HF medications if they were treated continuously for at least 6 months following their initial assessment, or an event occurred within the first 6 months while patients were continuously treated. If the medication was stopped during the first 6 months, then patients were classed as not taking the medication. The initial survival analysis was performed on the whole study population using a multivariable Cox proportional hazards regression adjusting for covariates selected *a priori* based on clinical relevance, association with HF medication treatment and association with survival [age, sex, ischaemic heart disease (IHD), diabetes mellitus, hypertension, atrial fibrillation, NAC disease stage, wild-type or hereditary ATTR-CA, interventricular septal thickness in diastole (IVSd), longitudinal strain, beta-blocker, ACEi/ARBs, and MRAs].

Propensity score (PS) matching is widely used to reduce confounding biases in observational studies. The PS is a score between 0 and 1 that reflects the likelihood of the patient receiving one of the HF medications of interest conditional on a set of variables, so that those with similar PSs are independent of these variables. Prior to PS matching, missing data were replaced using single imputation, whereby missing values of numerical variables were replaced by the relevant median, and missing values of categorical variables were replaced by the relevant mode, to overcome potential bias introduced by excluding patients with missing data. In order to compare two particular HF medications, a PS for each individual was determined using all the aforementioned variables, apart from the HF medications being assessed. After finding the area of common support (in which the histograms of the PSs overlapped), the patients were then matched on the basis of their PSs in the two medication groups in a 1:1 ratio using the nearest neighbour approach without replacement and calliper width equal to 0.20 times the standard deviation of the logit of the PSs. The adequacy of matching was verified by ensuring that the standardized differences between groups were <0.10 for all variables used to create the PS. A Cox proportional hazards regression model was then applied using the matched groups to compare the effect on survival of the two medications of interest. Additional PS-matched analyses specified *a priori* were carried out in the subgroup of patients with a left ventricular ejection fraction (LVEF) ≤40% and the subgroup of patients with a LVEF >40% (based on the guideline definition for HF with reduced ejection fraction being a LVEF ≤40%).^19^ Kaplan–Meier curves were constructed with statistical significance being assessed with a log-rank test. Significant results were followed by sensitivity analyses to assess whether these results could be replicated; firstly using an ‘intention to treat’ approach whereby patients were classed as treated, or not treated based on their treatment status at diagnosis (rather than over the first 6 months), secondly without censoring patients for the start date of clinical trials or disease-modifying therapy, and lastly analysing the medication use as a time-varying exposure. Statistical significance was defined as *P* < .05.

## Results

We identified 2371 patients diagnosed with ATTR-CA. The population compromised 1840 (77.6%) with wild-type ATTR-CA and 531 (22.4%) with hereditary ATTR-CA. The mean age of patients was 77.5 ± 7.3 years, and 90.0% were men. About two-thirds of patients were in New York Heart Association (NYHA) classes I and II, the median NT-proBNP was 2925 ng/L, and the mean LVEF was 48.2% [531 (22.4%) had a LVEF ≤40%). Most patients were in NAC stages 1 (45.8%) or 2 (36.0%). Approximately half of the patients had concomitant atrial fibrillation/flutter, and 54.2% had an estimated glomerular filtration rate (eGFR) <60 mL/min/1.73 m^2^. Overall, 1955 patients (82.4%) were treated with a diuretic. In most cases (76.8% patients) a loop diuretic was prescribed either alone or in combination (*Table 1*). A total of 467 (19.7%) patients were enrolled into clinical trials, or treated with disease-modifying therapy (clinical trials, *n* = 377; tafamidis, *n* = 90). These patients were younger and had a milder cardiac phenotype at diagnosis than the rest of the study population (see Supplementary data online, *Table S1*).

**Table 1:** Baseline characteristics and echocardiographic parameters for the overall population, and for patients separated into those with a LVEF >40% and a LVEF ≤40%

Baseline characteristics	Overall study population (*n* = 2371)	Patients with a LVEF >40% (*n* = 1840)	Patients with a LVEF ≤40% (*n* = 531)	*P*-value
Age	77.5 ± 7.3	77.6 ± 7.3	76.9 ± 7.3	.035
Sex (male)	2110 (90.0%)	1637 (89.0%)	473 (89.1%)	.943
Ethnicity				<.001
Caucasian	1893 (79.8%)	1525 (82.9%)*	368 (69.3%)	
Afro-Caribbean	444 (18.7%)	288 (15.7%)*	156 (29.4%)	
Asian	22 (0.9%)	18 (1.0%)	4 (0.8%)	
Other	12 (0.5%)	9 (0.5%)	3 (0.6%)	
wtATTR	1840 (77.6%)	1487 (80.8%)	353 (66.5%)	<.001
hATTR	531 (22.4%)	353 (19.2%)	178 (33.5%)	<.001
AF/flutter	1223 (51.6%)	937 (50.9%)	286 (53.9%)	.233
IHD	476 (20.1%)	374 (20.3%)	102 (19.2%)	.571
Diabetes mellitus	374 (15.8%)	273 (14.8%)	101 (19.0%)	.020
Hypertension	828 (34.9%)	631 (34.3%)	197 (37.1%)	.232
Stroke/TIA	109 (4.6%)	182 (9.9%)	76 (14.3%)	.004
CKD stages 3–5	1288 (54.3%)	953 (51.8%)	335 (63.1%)	<.001
Cardiac devices				
PPM	214 (9.0%)	173 (9.4%)	41 (7.7%)	.234
ICD	46 (1.9%)	29 (1.6%)	17 (3.2%)	.017
CRT-D	23 (1.0%)	14 (0.8%)	9 (1.7%)	.053
CRT-P	37 (1.6%)	24 (1.3%)	13 (2.4%)	.061
Heart failure severity				
NYHA class				<.001
1	317 (13.4%)	280 (15.2%)*	37 (7.0%)	
2	1387 (58.5%)	1093 (59.4%)*	294 (55.4%)	
3	435 (18.3%)	279 (15.2%)*	156 (29.4%)	
4	30 (1.3%)	19 (1.0%)	11 (2.1%)	
Missing	202	169	33	
NAC stage				<.001
1	1086 (45.8%)	926 (50.3%)*	160 (30.1%)	
2	853 (36.0%)	613 (33.3%)*	240 (45.2%)	
3	395 (16.7%)	266 (14.5%)*	129 (24.3%)	
Missing	37	35	2	
NT-proBNP (ng/L)	2925 (1530–5321)	2597 (1394–4786)	4123 (2484–7201)	<.001
eGFR (mL/min/1.73 m²)	58 (46–71)	59 (47–72)	54 (43–66)	<.001
6-Min walk test (meters)	347 (247–430)	354 (256–436)	322 (216–407)	<.001
6-Min walk test (% predicted)	71.2 ± 26.5	72.9 ± 25.7	64.7 ± 28.3	<.001
Systolic blood pressure (mmHg)	125.1 ± 21.4	126.3 ± 22.1	121.3 ± 18.7	<.001
Diastolic blood pressure (mmHg)	74.4 ± 12.7	73.9 ± 12.8	75.9 ± 12.4	.020
Heart rate (b.p.m.)	72.2 ± 13.7	71.1 ± 13.2	75.8 ± 14.4	<.001
Echocardiographic parameters				
IVSd (mm)	16.9 ± 2.4	16.9 ± 2.4	17.0 ± 2.4	.321
PWTd (mm)	16.3 ± 2.5	16.3 ± 2.5	16.4 ± 2.6	.220
MWT (mm)	17.1 ± 2.4	17.1 ± 2.4	17.2 ± 2.4	.326
Left atrial area (cm^2^)	26.2 ± 5.5	26.1 ± 5.5	26.6 ± 6.4	.069
Right atrial area (cm^2^)	24.5 ± 6.5	23.9 ± 6.4	26.3 ± 6.4	<.001
Stroke volume (mL)	37.3 ± 13.9	39.9 ± 13.8	29.4 ± 10.9	<.001
Simpson’s biplane LVEF (%)	48.2 ± 10.6	52.7 ± 7.2	33.6 ± 5.3	<.001
Longitudinal strain (%)	−10.8 ± 3.6	−11.7 ± 3.5	−8.1 ± 2.6	<.001
TAPSE (mm)	15.1 ± 4.9	15.9 ± 4.9	12.6 ± 3.5	<.001
E/e′	16.8 ± 6.4	16.5 ± 6.2	17.8 ± 7.0	<.001
Medications				
Beta-blockers	1313 (55.4%)	971 (52.8%)	342 (64.4%)	<.001
ACEi/ARBs	1362 (57.4%)	1041 (56.6%)	321 (60.5%)	.112
MRAs	925 (39.0%)	673 (36.6%)	252 (47.5%)	<.001
Loop diuretics	1808 (76.8%)	1357 (74.3%)	451 (85.3%)	<.001

Patients with hATTR-CA had the following variants: p.(Val142Ile) = 392, p.(Thr80Ala) = 93, p.(Ile127Val) = 12, p.(Ile88Leu) = 6, p.(Ser97Tyr) = 6, p.(Glu62Asp) = 4, p.(Glu109Lys) = 3, p.(Gly26Ser) = 3, p.(Val40Ile) = 2, p.(Val50Met) = 2, p.(Ala56Pro) = 1, p.(Asp58Tyr) = 1, p.(Asp58Val) = 1, p.(Asp59Val) = 1, p.(Glu74Gln) = 1, p.(Glu74Gly) = 1, p.(Glu74Leu) = 1, and p.(Phe64Leu) = 1.

AF, atrial fibrillation; IHD, ischaemic heart disease; TIA, transient ischaemic attack; CKD, chronic kidney disease; PPM, permanent pacemaker; ICD, implantable cardioverter defibrillator; CRT-D, cardiac resynchronization therapy defibrillator; CRT-P, cardiac resynchronization therapy pacemaker; NYHA, New York Heart Association; NAC, National Amyloidosis Centre; NT-proBNP, N-terminal pro B-type natriuretic peptide; eGFR, estimated glomerular filtration rate; IVSd, interventricular septum in diastole; PWTd, posterior wall thickness in diastole; MWT, maximal wall thickness; LVEF, left ventricular ejection fraction; ACEi, angiotensin-converting enzyme inhibitor; ARB, angiotensin II receptor blocker; MRA, mineralocorticoid receptor antagonist.

*P* < .05.

### Prescription pattern of heart failure medications

#### Beta-blockers

A total of 1313 (55.4%) patients were treated with beta-blockers (64.4% in patients with a LVEF ≤40%) at diagnosis. Those treated with beta-blockers had a higher prevalence of IHD, diabetes mellitus, and atrial fibrillation compared to patients not receiving this type of treatment. Those treated with beta-blockers had a more severe cardiac phenotype, with a worse functional capacity as evidenced by NYHA class and 6-min walk test (6MWT), and a higher NAC disease stage [a greater proportion of patients had stage 3 (severe) disease]. The median NT-proBNP among patients treated with beta-blockers was significantly higher, while median eGFR was significantly lower than patients not receiving beta-blockers. Patients treated with beta-blockers had a larger bi-atrial size, lower LVEF, lower tricuspid annular plane systolic excursion (TAPSE), and worse longitudinal strain than those not receiving this type of treatment.

#### Renin-angiotensin system blockers

A total of 1362 (57.4%) patients were treated with an ACEi or ARB (60.5% in patients with a LVEF ≤40%) at diagnosis. As for beta-blockers, those treated with ACEi/ARBs had a higher prevalence of IHD, diabetes mellitus, and atrial fibrillation compared to patients not receiving this type of treatment. In addition, patients treated with an ACEi/ARB were more likely to have hypertension than patients not receiving this type of treatment. Those treated with ACEi/ARBs had a severe cardiac phenotype, with a higher NYHA class and NAC disease stage, and a higher proportion of patients having chronic kidney disease stages 3–5 than patients not receiving ACEi/ARBs. Patients treated with ACEi/ARBs had a larger bi-atrial size, lower LVEF, and worse longitudinal strain than those not receiving this type of treatment.

#### Mineralocorticoid receptor antagonists

A total of 925 (39.0%) patients were treated with an MRA (47.5% in patients with a LVEF ≤40%) at diagnosis. Those treated with MRAs had a higher prevalence of diabetes mellitus and atrial fibrillation but, unlike beta-blocker and ACEi/ARB treatment, patients treated with an MRA did not have more IHD. Those treated with MRAs had a more severe cardiac phenotype, with a worse functional capacity as evidenced by NYHA class and 6MWT, and a higher NAC disease. The median NT-proBNP among patients treated with MRAs was significantly higher, while median eGFR was significantly lower than patients not receiving MRAs. Patients treated with MRAs had a larger right atrial area, lower stroke volume, lower LVEF, lower TAPSE, higher E/e′, and worse longitudinal strain than those not receiving this type of treatment (*Table 2*).

**Table 2:** Baseline characteristics and echocardiographic parameters for patients treated with heart failure medications compared to patients not treated with heart failure medications

Variables	Patients with cardiac ATTR amyloidosis split by treatment with beta-blockers			Patients with cardiac ATTR amyloidosis split by treatment with ACEi/ARBs			Patients with cardiac ATTR amyloidosis split by treatment with MRAs		
	Patients treated with beta-blockers (*n* = 1313, 55.4%)	Patients not treated with beta-blockers (*n* = 1058, 44.6%)	*P*-value	Patients treated with ACEi/ARB (*n* = 1362, 57.4%)	Patients not treated with ACEi/ARB (*n* = 1009, 42.6%)	*P*-value	Patients treated with MRAs (*n* = 925, 39.0%)	Patients not treated with MRAs (*n* = 1446, 61.0%)	*P*-value
Baseline characteristics									
Age	77.4 ± 6.9	77.6 ± 7.7	.456	77.4 ± 6.7	77.6 ± 8.0	.546	76.9 ± 6.9	77.9 ± 7.5	.001
Sex (male)	1172 (89.3%)	938 (88.7%)	.641	1224 (89.9%)	886 (87.8%)	.113	824 (89.1%)	1286 (88.9%)	.912
Ethnicity			.016			.040			<.001
Caucasian	1030 (78.4%)	863 (81.6%)		1069 (78.5%)	824 (81.7%)		695 (75.1%)*	1198 (82.8%)	
Afro-Caribbean	269 (20.5%)*	175 (16.5%)		278 (20.4%)*	166 (16.5%)		215 (23.2%)*	229 (15.8%)	
Asian	11 (0.8%)	11 (1.0%)		9 (0.7%)	13 (1.3%)		9 (1.0%)	13 (0.9%)	
Other	3 (0.2%)*	9 (0.9%)		6 (0.4%)	6 (0.6%)		6 (0.6%)	6 (0.4%)	
wtATTR	1021 (77.8%)	819 (77.4%)	.839	1067 (78.3%)	773 (76.6%)	.318	686 (74.2%)	1154 (79.8%)	<.001
hATTR	292 (22.2%)	239 (22.6%)	.839	295 (21.7%)	236 (23.4%)	.318	239 (25.8%)	292 (20.2%)	<.001
AF/flutter	755 (57.5%)	468 (44.2%)	<.001	728 (53.5%)	495 (49.1%)	.034	515 (55.7%)	708 (49.0%)	.001
IHD	300 (22.8%)	176 (16.6%)	<.001	301 (22.1%)	175 (17.3%)	.004	198 (21.4%)	278 (19.2%)	.196
Diabetes mellitus	241 (18.4%)	133 (12.6%)	<.001	249 (18.2%)	125 (12.4%)	<.001	168 (18.2%)	206 (14.2%)	.011
Hypertension	479 (36.5%)	349 (33.0%)	.079	549 (40.3%)	279 (27.7%)	<.001	335 (36.2%)	493 (34.1%)	.290
Stroke/TIA	137 (10.4%)	121 (11.4%)	.436	143 (10.5%)	115 (11.4%)	.487	97 (10.5%)	161 (11.1%)	.670
CKD stages 3–5	797 (60.7%)	491 (46.4%)	<.001	774 (56.8%)	514 (50.9%)	.004	573 (61.9%)	715 (49.4%)	<.001
Cardiac devices									
PPM	118 (9.0%)	96 (9.1%)	.942	138 (10.1%)	76 (7.5%)	.029	95 (10.3%)	119 (8.2%)	.091
ICD	32 (2.4%)	14 (1.3%)	.051	28 (2.1%)	18 (1.8%)	.635	22 (2.4%)	24 (1.7%)	.216
CRT-D	15 (1.1%)	8 (0.8%)	.340	17 (1.2%)	6 (0.6%)	.108	13 (1.4%)	10 (0.7%)	.084
CRT-P	21 (1.6%)	16 (1.5%)	.865	23 (1.7%)	14 (1.4%)	.559	21 (2.3%)	16 (1.1%)	.026
Heart failure severity									
NYHA class			<.001			<.001			<.001
1	135 (10.3%)*	182 (17.2%)		143 (10.5%)*	174 (17.2%)		87 (9.4%)*	230 (15.9%)	
2	755 (57.5%)	632 (59.7%)		795 (56.2%)	592 (58.7%)		555 (60.0%)	832 (57.5%)	
3	286 (21.8%)*	149 (14.1%)		275 (20.2%)*	160 (15.9%)		219 (23.7%)*	216 (14.9%)	
4	18 (1.4%)	12 (1.1%)		20 (1.5%)	10 (1.0%)		15 (1.6%)	15 (1.0%)	
Missing	119	83		129	73		49	153	
NAC stage			<.001			.047			<.001
1	524 (40.0%)*	562 (52.5%)		607 (44.6%)	479 (47.4%)		379 (41.0%)*	707 (48.9%)	
2	522 (39.8%)*	331 (31.1%)		519 (38.1%)*	334 (33.1%)		369 (39.9%)*	484 (33.5%)	
3	254 (19.3%)*	141 (13.3%)		217 (15.9%)	178 (17.6%)		167 (18.1%)	228 (15.8%)	
Missing	13	24		19	18		10	27	
NT-proBNP (ng/L)	3369 (1886–5912)	2391 (1285–4540)	<.001	2999 (1591–5274)	2850 (1479–5381)	.095	3136 (1806–5420)	2732 (1433–5248)	<.001
eGFR (mL/min/1.73 m²)	56 (45–69)	62 (48–75)	<.001	58 (46–70)	60 (46–73)	.139	55 (45–68)	60 (47–74)	<.001
6-Min walk test (meters)	343 (230–422)	358 (268–442)	.001	349 (242–428)	335 (253–433)	.985	336 (230–424)	358 (266–437)	.004
6-Min walk test (% predicted)	68.0 ± 26.6	75.7 ± 25.7	<.001	71.3 ± 26.4	71.0 ± 26.6	.853	67.9 ± 26.4	74.0 ± 26.3	<.001
Systolic blood pressure (mmHg)	123.7 ± 20.2	127.0 ± 22.9	<.001	124.8 ± 20.8	125.6 ± 22.3	.028	121.8 ± 19.2	127.3 ± 22.6	<.001
Diastolic blood pressure (mmHg)	74.0 ± 12.5	74.9 ± 13.0	<.001	74.0 ± 13.3	75.0 ± 13.5	.004	72.8 ± 11.5	75.5 ± 14.1	<.001
Heart rate (b.p.m.)	71.2 ± 14.0	73.5 ± 13.1	<.001	71.9 ± 13.5	72.7 ± 13.9	.230	72.0 ± 14.1	72.4 ± 13.4	.471
Echocardiographic parameters									
IVSd (mm)	17.00 ± 2.4	16.9 ± 2.5	.672	17.0 ± 2.5	16.8 ± 2.4	.051	17.1 ± 2.4	16.8 ± 2.5	.015
PWTd (mm)	16.4 ± 2.5	16.3 ± 2.6	.761	16.4 ± 2.5	16.3 ± 2.5	.412	16.5 ± 2.5	16.3 ± 2.5	.014
MWT (mm)	17.1 ± 2.4	17.1 ± 2.5	.677	17.2 ± 2.4	17.0 ± 2.4	.061	17.3 ± 2.3	17.0 ± 2.5	.020
Left atrial area (cm^2^)	26.7 ± 5.4	25.6 ± 6.4	<.001	26.54 ± 5.6	25.78 ± 5.4	.008	26.5 ± 5.4	26.0 ± 5.7	.113
Right atrial area (cm^2^)	25.1 ± 6.4	23.5 ± 6.4	<.001	24.9 ± 6.6	23.8 ± 6.6	<.001	25.1 ± 5.4	24.0 ± 6.4	<.001
Stroke volume (mL)	36.6 ± 13.9	38.3 ± 14.0	.023	37.7 ± 14.5	36.6 ± 13.0	.126	35.9 ± 13.0	38.5 ± 14.6	<.001
LVEF (%)	47.1 ± 10.7	49.5 ± 10.4	<.001	47.7 ± 10.6	48.8 ± 10.7	.013	46.4 ± 10.6	49.3 ± 10.5	<.001
LVEF ≤40%	342 (26.0%)	189 (17.9%)	<.001	321 (23.6%)	210 (20.8%)	.112	252 (27.2%)	279 (19.3%)	<.001
Longitudinal strain (%)	−10.6 ± 3.5	−11.1 ± 3.8	<.001	−10.6 ± 3.5	−11.0 ± 3.7	.014	−10.2 ± 3.3	−11.2 ± 3.8	<.001
TAPSE (mm)	14.7 ± 4.8	15.5 ± 5.0	.002	15.0 ± 4.7	15.2 ± 5.2	.342	14.6 ± 4.8	15.5 ± 5.0	<.001
E/e′	16.7 ± 6.4	16.7 ± 6.5	.567	16.9 ± 6.2	16.7 ± 6.7	.640	17.2 ± 6.5	16.3 ± 6.3	.036

BSA, body surface area; AF, atrial fibrillation; IHD, ischaemic heart disease; TIA, transient ischaemic attack; CKD, chronic kidney disease; PPM, permanent pacemaker; ICD, implantable cardioverter defibrillator; CRT-D, cardiac resynchronization therapy defibrillator; CRT-P, cardiac resynchronization therapy pacemaker; NYHA, New York Heart Association; NAC, National Amyloidosis Centre; NT-proBNP, N-terminal pro B-type natriuretic peptide; eGFR, estimated glomerular filtration rate; IVSd, interventricular septum in diastole; PWTd, posterior wall thickness in diastole; MWT, maximal wall thickness; LVEF, left ventricular ejection fraction.

*P* < .05.

#### Combination heart failure therapy

A total of 417 (17.6%) patients were treated with all three classes of HF medications (beta-blocker, ACEi/ARB, and MRA) at diagnosis, 804 (33.9%) were treated with a combination of two classes of HF medications, 741 (31.3%) were treated with one of the three classes of HF medications, and 409 (17.2%) were not treated with any prognostic HF medications. The most frequent combination of two HF medications was a beta-blocker and ACEi/ARB in 454 (56.5%) patients, followed by a beta-blocker and MRA in 180 (22.4%) patients, and an ACEi/ARB and MRA in 170 (21.1%) patients. Those treated with more HF medications had a higher prevalence of IHD, diabetes mellitus, and atrial fibrillation. They had more severe HF, with a worse functional status, as evidenced by NYHA class and 6MWT, and a higher NAC disease stage, and a higher proportion of patients having chronic kidney disease stages 3–5. Patients treated with more HF medications had a larger left ventricular wall thickness, larger bi-atrial size, and worse biventricular systolic function (reflected in a lower TAPSE, LVEF, and worse longitudinal strain), and there was a greater use of HF medications in patients with a LVEF ≤40%(*Table 3*).

**Table 3:** Baseline characteristics and echocardiographic parameters for the overall population, separated by the number of heart failure medications patients were treated with

Baseline characteristics	Not treated with HF medications (*n* = 409, 17.2%)	Treated with one HF medication (*n* = 741, 31.3%)	Treated with two HF medications (*n* = 804, 33.9%)	Treated with three HF medications (*n* = 417, 17.6%)	*P*-value
Age	76.8 ± 9.0*	78.5 ± 6.9*****	77.6 ± 6.6******	76.1 ± 6.9	<.001
Sex (male)	359 (87.8%)	653 (88.1%)	727 (90.4%)	371 (89.0%)	.412
Ethnicity					<.001
Caucasian	336 (82.2%)***	615 (83.0%)*****	647 (80.5%)******	295 (70.7%)	
Afro-Caribbean	66 (16.1%)***	113 (15.2%)*****	146 (18.2%)******	119 (28.5%)	
Asian	3 (0.7%)	10 (1.3%)	8 (1.0%)	1 (0.2%)	
Other	4 (1.0%)	3 (0.4%)	3 (0.4%)	2 (0.5%)	
wtATTR	298 (72.9%)*,***	607 (81.9%)*****	638 (79.4%)******	297 (71.2%)	<.001
hATTR	111 (27.1%)*,***	134 (18.1%)*****	166 (20.6%)******	120 (28.8%)	<.001
AF/flutter	145 (35.5%)*,**,***	389 (52.5%)	458 (57.0%)	231 (55.4%)	<.001
IHD	54 (13.2%)**,***	142 (19.2%)	183 (22.8%)	97 (23.3%)	<.001
Diabetes mellitus	41 (10.0%)**,***	104 (14.0%)*****	133 (16.5%)	96 (15.8%)	<.001
Hypertension	92 (22.5%)*,**,***	276 (37.2%)	293 (36.4%)	167 (40.0%)	<.001
Stroke/TIA	50 (12.2%)	78 (10.5%)	91 (11.3%)	39 (9.4%)	.568
CKD stages 3–5	151 (36.9%)*,**,***	394 (53.2%)*****	479 (59.6%)	264 (63.3%)	<.001
Cardiac devices					
PPM	30 (7.4%)	55 (7.4%)	91 (11.3%)	378 (9.1%)	.031
ICD	6 (1.5%)	11 (1.5%)	16 (2.0%)	13 (3.1%)	.227
CRT-D	1 (0.2%)	6 (0.8%)	9 (1.1%)	7 (1.7%)	.187
CRT-P	1 (0.2%)	13 (1.8%)	17 (2.1%)	6 (1.4%)	.092
Heart failure severity					
NYHA class					<.001
1	105 (25.7%)*,**,***	92 (12.4%)*****	87 (10.8%)	33 (7.9%)	
2	251 (61.4%)	412 (55.6%)	479 (59.6%)******	245 (58.8%)	
3	50 (12.2%)***	120 (16.2%)*****	135 (16.8%)******	130 (31.2%)	
4	3 (0.7%)	9 (1.2%)	10 (1.2%)	8 (1.9%)	
Missing	0	108	93	1	
NAC stage					<.001
1	214 (52.3%)*,**,***	346 (46.7%)	333 (41.4%)	166 (39.8%)	
2	133 (32.5%)**,***	247 (33.3%)*****	316 (39.3%)	177 (42.4%)	
3	44 (10.8%)*,**	135 (18.2%)	145 (18.0%)	71 (17.0%)	
Missing	18	13	10	3	
NT-proBNP (ng/L)	2142 (1038–4224)*,**,***	2899 (1517–5259)****	3254 (1705–5785)	3201 (1958–5454)	<.001
eGFR (mL/min/1.73 m²)	66 (52–79)*,**,***	59 (45–71)	56 (45–70)	55 (46–66)	<.001
6-Min walk test (meters)	368 (276–447)***	350 (264–437)	345 (241–431)	332 (221–414)	.015
6-Min walk test (% predicted)	75.8 ± 26.8***	74.0 ± 26.0*****	70.7 ± 25.9	65.4 ± 27.0	<.001
Systolic blood pressure (mmHg)	127.6 ± 25.6**,***	128.0 ± 20.5****,*****	123.2 ± 20.9	121.9 ± 18.8	<.001
Diastolic blood pressure (mmHg)	75.3 ± 14.6**,***	76.0 ± 12.5****,*****	73.6 ± 12.6	72.5 ± 11.4	<.001
Heart rate (b.p.m.)	73.8 ± 13.0**	72.7 ± 13.3	71.5 ± 14.2	71.3 ± 13.6	.008
Echocardiographic parameters					
IVSd (mm)	16.6 ± 2.5**	16.9 ± 2.5	17.1 ± 2.5	16.9 ± 2.2	.011
PWTd (mm)	16.0 ± 2.6**	16.4 ± 2.4	16.5 ± 2.5	16.2 ± 2.6	.007
MWT (mm)	16.8 ± 2.5**	17.1 ± 2.4	17.3 ± 2.5	17.1 ± 2.2	.008
Left atrial area (cm^2^)	25.1 ± 5.5**,***	25.9 ± 5.7	26.6 ± 5.4	26.8 ± 5.4	<.001
Right atrial area (cm^2^)	22.6 ± 6.2**,***	23.7 ± 6.5****,*****	25.3 ± 6.1	25.4 ± 6.7	<.001
Stroke volume (mL)	38.4 ± 13.8	38.2 ± 14.2	36.8 ± 13.8	36.3 ± 13.8	.158
LVEF (%)	50.4 ± 10.2**,***	49.0 ± 10.5*****	48.0 ± 10.8******	45.1 ± 10.2	<.001
LVEF ≤40%	62 (15.2%)**,***	155 (20.9%)	182 (22.6%)******	132 (31.7%)	<.001
Longitudinal strain (%)	−11.6 ± 4.0**,***	−11.2 ± 3.7****	−10.4 ± 3.4	−10.1 ± 3.3	<.001
TAPSE (mm)	15.9 ± 5.1**,***	15.4 ± 5.0	14.7 ± 5.0	14.6 ± 4.4	.001
E/e′	16.5 ± 6.9	16.8 ± 6.4	16.8 ± 6.0	17.1 ± 6.6	.707

HF, heart failure; AF, atrial fibrillation; IHD, ischaemic heart disease; TIA, transient ischaemic attack; CKD, chronic kidney disease; PPM, permanent pacemaker; ICD, implantable cardioverter defibrillator; CRT-D, cardiac resynchronization therapy defibrillator; CRT-P, cardiac resynchronization therapy pacemaker; NYHA, New York Heart Association; NAC, National Amyloidosis Centre; NT-proBNP, N-terminal pro B-type natriuretic peptide; eGFR, estimated glomerular filtration rate; IVSd, interventricular septum in diastole; PWTd, posterior wall thickness in diastole; MWT, maximal wall thickness; LVEF, left ventricular ejection fraction.

*P* < .05 for no HF medications vs. one HF medication.

*P* < .05 for no HF medications vs. two HF medications.

*P* < .05 for no HF medications vs. three HF medications.

*P* < .05 for one HF medication vs. two HF medications.

*P* < .05 for one HF medication vs. three HF medications.

*P* < .05 for two HF medications vs. three HF medications.

### Doses of heart failure medications and discontinuation rates

#### Beta-blockers

Of the 1313 patients treated with beta-blockers, over half were treated with ≤25% of the target dose for HF (*n* = 829, 63.1%).^19^ The most commonly prescribed beta-blocker was bisoprolol (*n* = 1164, 88.7%), with the majority of patients treated with ≤2.5 mg per day (*n* = 721, 61.9%). Only 75 (5.7%) patients had the target beta-blocker dose prescribed, most of which had atrial fibrillation (*n* = 58, 77.3%). The overwhelming majority of the study population (*n* = 1266, 96.4%) and all patients with a LVEF ≤40% (*n* = 342, 100.0%) were treated with beta-blockers approved for HF with reduced ejection fraction. During follow-up 285 (21.7%) patients had their beta-blocker discontinued [median duration to discontinuation: 14.1 (6.8–28.9) months], and 117 (8.9%) had their beta-blocker dose reduced [median duration to reduction: 15.7 (7.4–34.5) months]. Patients who discontinued beta-blocker treatment had a lower blood pressure and heart rate than those who continued treatment. Only 63 (4.8%) patients had their beta-blocker dose increased, of which only 8 patients eventually had the target dose prescribed. During follow-up, 55 patients were initiated on beta-blockers, and the majority were treated with ≤25% of the target dose (*n* = 44, 80.0%), of which 4 (7.2%) had their beta-blocker subsequently discontinued.

#### Renin-angiotensin system blockers

Of the 1362 patients treated with ACEi/ARBs, over half were treated with ≤37.5% of the target dose (*n* = 728, 53.5%).^19^ The most commonly prescribed ACEi/ARB was ramipril (*n* = 701, 51.4%), with the majority of patients treated with ≤2.5 mg per day (*n* = 354, 50.5%). Only 158 (11.6%) patients were treated with the target ACEi/ARB dose. During follow-up 448 (32.9%) patients had their ACEi/ARB discontinued [median duration to discontinuation: 14.4 (6.9–26.8) months], and 77 (5.7%) had their ACEi/ARB dose reduced [median duration to reduction: 14.2 (7.4–26.6) months]. Patients who discontinued ACEi/ARB treatment had a lower blood pressure than those who continued treatment (see Supplementary data online, *Table S2*). Only 35 (2.6%) patients had their ACEi/ARB dose increased, of which only 3 patients were treated with the target dose. During follow-up, 41 patients were initiated on ACEi/ARBs, and the majority were treated with ≤37.5% of the target dose (*n* = 26, 63.4%) of which 8 (19.5%) had their ACEi/ARB subsequently discontinued.

#### Mineralocorticoid receptor antagonists

Of the 925 patients treated with MRAs, 742 (80.2%) were treated with spironolactone, and 183 (19.3%) were treated with eplerenone. The most commonly prescribed dose of both drugs was 25 mg (*n* = 657, 71.0%), followed by 50 mg (*n* = 79, 8.5%). During follow-up 69 (7.5%) patients had their MRAs discontinued [median duration to discontinuation: 12.5 (7.9–24.9) months], and 31 (3.4%) had their MRA dose reduced [median duration to reduction: 14.1 (7.9–24.9) months]. Only 77 (8.3%) patients had the dose of their MRA increased, of which 53 were prescribed with 50 mg. During follow-up, 158 patients were initiated on MRAs, and the majority were treated with ≥25 mg (*n* = 129, 81.6%), of which only 5 (3.2%) had their MRA subsequently discontinued.

### Association between heart failure medication classes and survival

In the overall population, median follow-up was 27.8 months (IQR: 10.6–51.3), and the death rate was 14.9 deaths per 100 patient-years (95% CI 13.9–15.9). There were 1274 patients classed as being treated with beta-blockers for the survival analysis, and the death rate was 14.8 deaths per 100 patient-years (95% CI 13.5–16.2). There were 1306 patients classed as being treated with ACEi/ARBs for the survival analysis, and the death rate was 15.0 deaths per 100 patient-years (95% CI 13.8–16.4). There were 915 patients classed as being treated with MRAs for the survival analysis, and the death rate was 14.6 deaths per 100 patient-years (95% CI 13.1–16.1).

#### Multivariable Cox regression model

In a multivariable Cox regression analysis with covariates age, sex, IHD, diabetes mellitus, hypertension, atrial fibrillation, NAC disease stage, wild-type or hereditary ATTR-CA, IVSd, longitudinal strain, beta-blocker, ACEi/ARB, and MRA, only 4 covariates (age, hATTR-CA, higher NAC disease stage, and worse longitudinal strain) were associated with a higher risk of mortality; and only one treatment [MRA: HR 0.82 (95% CI 0.71–0.94), *P* = .004] was convincingly associated with a lower risk of mortality (see Supplementary data online, *Table S3*).

#### Propensity score-matched analyses

**Figure 1:**
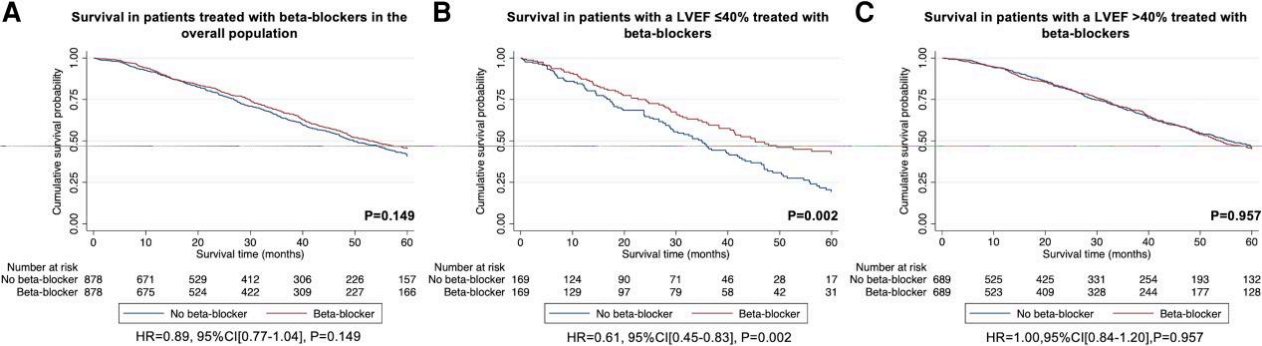
Kaplan–Meier curves comparing survival in patients treated with beta-blockers to patients not treated with beta-blockers followed by a Cox proportional hazards regression analysis: (*A*) treatment with beta-blockers vs. no treatment with beta-blockers in the overall population, (*B*) treatment with beta-blockers vs. no treatment with beta-blockers in patients with a LVEF ≤40%, (*C*) treatment with beta-blockers vs. no treatment with beta-blockers in patients with a LVEF >40%

To minimize the potential selection bias inherent with the baseline treatment of HF medications we also performed PS-matched cohort analyses to assess the association between treatment with each HF medication and survival. Missing data were imputed for NAC stage in 37 patients, IVSd in 115 patients, and longitudinal strain in 296 patients. The remaining variables did not have any missing data. The PS-matched cohort constructed to assess the association between treatment with beta-blockers and risk of mortality comprised of 1756 patients (878 treated with beta-blockers vs. 878 not treated with beta-blockers) and did not provide convincing evidence for a difference in the risk of mortality between the two groups [HR 0.89 (95% CI 0.77–1.04), *P* = .149], although the 95% CI of the estimate was wide and did not exclude clinically important effects (see Supplementary data online, *Table S4*). A second PS-matched cohort was constructed to assess the association between treatment with beta-blockers and risk of mortality in patients with a LVEF ≤40%. This comprised of 338 patients (169 treated with beta-blockers vs. 169 not treated with beta-blockers), and demonstrated a 39% lower risk of mortality in patients treated with beta-blockers [HR 0.61 (95% CI 0.45–0.83), *P* = .002] (see Supplementary data online, *Table S5*). These findings were confirmed with sensitivity analysis, utilizing an ‘intention to treat’ approach [HR 0.58 (95% CI 0.42–0.81), *P* = .001], and whereby patients were no longer censored for the start date of clinical trials and disease modifying therapy [HR 0.63 (95% CI 0.47–0.85), *P* = .003], and where beta-blocker treatment was analysed as a time-varying exposure [HR 0.51 (95% CI 0.37–0.71), *P* < .001]. Following exclusion of patients with coexistent IHD and their corresponding pairs, repeat analysis confirmed a lower risk of mortality in patients with a LVEF ≤40% treated with beta-blockers [HR 0.56 (95% CI 0.38–0.83), *P* = .003]. A third PS-matched cohort was constructed to assess the association between treatment with beta-blockers and risk of mortality in patients with a LVEF >40%. This comprised of 1378 patients (689 treated with beta-blockers vs. 689 not treated with beta-blockers) and did not provide convincing evidence for a difference in the risk of mortality between the two groups [HR 1.00 (95% CI 0.84–1.20), *P* = .957], although the estimate was imprecise (*Figure 1* and Supplementary data online, *Table S6*).

**Figure 2:**
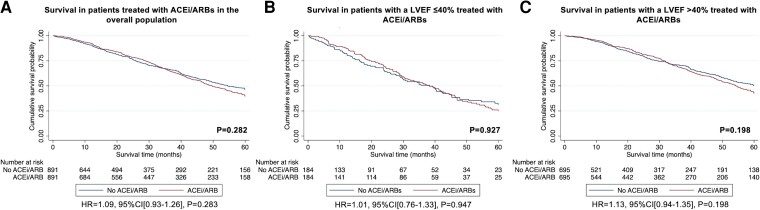
Kaplan–Meier curves comparing survival in patients treated with ACEi/ARBs to patients not treated with ACEi/ARBs followed by a Cox proportional hazards regression analysis: (*A*) treatment with ACEi/ARBs vs. no treatment with ACEi/ARBs in the overall population, (*B*) treatment with ACEi/ARBs vs. no treatment with ACEi/ARBs in patients with a LVEF ≤40%, (*C*) treatment with ACEi/ARBs vs. no treatment with ACEi/ARBs in patients with a LVEF >40%

The PS-matched cohort constructed to assess the association between treatment with ACEi/ARBs and the risk of mortality comprised of 1782 patients (891 treated with ACEi/ARBs vs. 891 not treated with ACEi/ARBs) and did not provide convincing evidence for a difference in the risk of mortality between the two groups [HR 1.09 (95% CI 0.93–1.26), *P* = .283] (see Supplementary data online, *Table S7*). A second PS-matched analysis was constructed to assess the association between treatment with ACEi/ARBs and the risk of mortality in patients with a LVEF ≤40%. This comprised of 368 patients (184 treated with ACEi/ARBs vs. 184 not treated with ACEi/ARBs) and did not provide convincing evidence for a difference in the risk of mortality between the two groups [HR 1.01 (95% CI 0.76–1.33), *P* = .947], although the estimates were imprecise (Supplementary data online, *Table S8*). A third PS-matched analysis was constructed to assess the association between treatment with ACEi/ARBs and the risk of mortality in patients with a LVEF >40%. This comprised of 1390 patients (695 treated with ACEi/ARBs vs. 695 not treated with ACEi/ARBs) and did not provide convincing evidence for a difference in the risk of mortality between the two groups [HR 1.13 (95% CI 0.94–1.35), *P* = .198] (*Figure 2* and Supplementary data online, *Table S9*).

**Figure 3:**
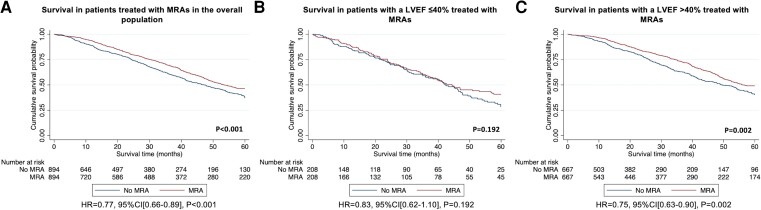
Kaplan–Meier curves comparing survival in patients treated with MRAs to patients not treated with MRAs followed by a Cox proportional hazards regression analysis: (*A*) treatment with MRAs vs. no treatment with MRAs in the overall population, (*B*) treatment with MRAs vs. no treatment with MRAs in patients with a LVEF ≤40%, (*C*) treatment with MRAs vs. no treatment with MRAs in patients with a LVEF >40%

The PS-matched cohort constructed to assess the association between treatment with MRAs and the risk of mortality comprised of 1788 patients (894 patients treated with MRAs vs. 894 patients not treated with MRAs) and demonstrated that there was a 23% lower risk of mortality in patients treated with MRAs [HR 0.77 (95% CI 0.66–0.89), *P* < .001] (see Supplementary data online, *Table S10*). These findings were confirmed with sensitivity analysis, utilizing an ‘intention to treat’ approach [HR 0.81 (95% CI 0.69–0.94), *P* = .006]; and whereby patients were no longer censored for the start date of clinical trials and disease modifying therapy [HR 0.78 (95% CI 0.67–0.90), *P* < .001], and where MRA treatment was analysed as a time-varying exposure [HR 0.81 (95% CI 0.69–0.94), *P* = .004]. A second PS-matched analysis was constructed to assess the association between treatment with MRAs and the risk of mortality in patients with a LVEF ≤40%. This comprised of 416 patients (208 patients treated with MRAs vs. 208 patients not treated with MRAs) and did not provide convincing evidence for a difference in the risk of mortality between the two groups [HR 0.83 (95% CI 0.62–1.10), *P* = .192], although the 95% CI of the estimate was wide and did not exclude clinically important effects (see Supplementary data online, *Table S11*). A third PS-matched analysis was constructed to assess the association between treatment with MRAs and the risk of mortality in patients with a LVEF >40%. This comprised of 1334 patients (667 treated with MRAs vs. 667 not treated with MRAs) and demonstrated that there was a 25% lower risk of mortality in patients treated with MRAs [HR 0.75 (95% CI 0.63–0.90), *P* = .002] (*Figure 3* and Supplementary data online, *Table S12*). These findings were confirmed with sensitivity analysis, utilizing an ‘intention to treat’ approach [HR 0.78 (95% CI 0.65–0.94), *P* = .008]; and whereby patients were no longer censored for the start date of clinical trials and disease modifying therapy [HR 0.79 (95% CI 0.66–0.94), *P* = .009], and where MRA treatment was analysed as a time-varying exposure [HR 0.77 (95% CI 0.65–0.93), *P* = .005].

#### Propensity score-matched analyses for combination therapy

In the overall population, a PS-matched cohort was constructed to compare the association with risk of mortality, between treatment with all three classes of HF medications (beta-blockers, ACEi/ARBs, and MRAs) and treatment with two classes of HF medications (beta-blockers and ACEi/ARBs). This comprised of 680 patients (340 treated with all three HF medications vs. 340 treated with beta-blockers and ACEi/ARBs) and demonstrated that there was a 37% lower risk of mortality in patients treated with all three HF medications [HR 0.63 (95% CI 0.49–0.80), *P* < .001] (see Supplementary data online, *Table S13*). These findings were confirmed with sensitivity analysis, utilizing an ‘intention to treat’ approach [HR 0.64 (95% CI 0.50–0.83), *P* < .001]; and whereby patients were no longer censored for the start date of clinical trials and disease modifying therapy [HR 0.65 (95% CI 0.51–0.82), *P* < .001], and where treatment was analysed as a time-varying exposure [HR 0.56 (95% CI 0.0.41–0.78), *P* = .001].

A PS-matched cohort was used to compare the association with risk of mortality, between treatment with two classes of HF medications (beta-blockers and ACEi/ARBs) and treatment with just ACEi/ARBs. This comprised of 558 patients (279 treated with beta-blockers and ACEi/ARBs vs. 279 treated with just ACEi/ARBs) and did not provide convincing evidence for a difference in the risk of mortality between the two groups [HR 1.06 (95% CI 0.81–1.39), *P* = .677], although the estimates were imprecise (see Supplementary data online, *Table S14* and Supplementary data online, *Figure S1*). Data on reasons for medication discontinuation and the association between survival and both medication dosage and medication discontinuation are presented in Supplementary data online, *Appendix S1*.

## Discussion

In this study we comprehensively evaluated the prescription pattern and discontinuation rates of HF medications in >2000 patients with ATTR-CA, and assessed the association between treatment with HF medications and the risk of mortality in these individuals. Our study demonstrated that: (i) patients with ATTR-CA and a severe cardiac phenotype were more commonly treated with HF medications; (ii) beta-blockers and ACEi/ARBs were generally prescribed in low doses and often discontinued, whereas in contrast, MRAs were rarely discontinued; and (iii) MRAs were independently associated with a lower risk of mortality in the overall population, and in patients with LVEF >40%; and low-dose beta-blockers were independently associated with a lower risk of mortality in patients with a LVEF ≤40% (*Structured Graphical Abstract*).

In the overall population of patients with ATTR-CA, a relatively low proportion were treated with beta-blockers (55.4%), ACEi/ARBs (57.4%), and MRAs (39.0%). Treatment with HF medications in patients with ATTR-CA appears to be driven by the presence of comorbidities and the severity of their cardiac disease. Heart failure medications were more commonly prescribed in patients with atrial fibrillation, diabetes mellitus, and chronic kidney disease. Beta-blockers and ACEi/ARBs are also more commonly prescribed in patients with IHD.^15,20^ Patients treated with conventional HF medications had more advanced cardiac disease as evidenced by worse functional capacity, a more severe NAC disease stage and lower indices of systolic function. Radial systolic impairment occurs in advanced ATTR-CA, and since the main evidence base for conventional HF medications is in patients with a LVEF ≤40%, the development of systolic impairment is likely to have contributed to greater use of HF medications in those with advanced cardiac disease.^6–12^

Beta-blockers and ACEi/ARBs were commonly discontinued, with over one-fifth of patients having their beta-blocker discontinued, and nearly one-third having their ACEi/ARB discontinued during follow-up. Beta-blocker intolerance may be exacerbated the underlying pathophysiology of ATTR-CA. In the context of a fixed stroke volume, caused by restrictive physiology, a higher heart rate is required to maintain cardiac output. The inability to augment stroke volume in response to the vasodilation may also contribute to the intolerance of ACEi/ARBs.^15,21^ In contrast, MRAs were rarely discontinued, with less than one-tenth having their MRA discontinued. This is probably related to the limited effect on blood pressure, compared with beta-blockers and ACEi/ARB, and their possible diuretic effect. The mainstay of symptom management in ATTR-CA has long been meticulous volume control, facilitated by high-dose loop diuretics. The MRAs may have a synergistic effect when utilized alongside loop diuretics and also increase potassium reabsorption, which is often needed when high doses of loop diuretics are utilized.^22^

In the current study, which represents the largest analysis of HF medications in patients with ATTR-CA to date, both regression-based and PS-matched analyses demonstrated that treatment with MRAs was independently associated with a lower risk of mortality in the overall ATTR-CA population; and PS-matched analysis demonstrated that low-dose beta-blockers were associated with a lower risk of mortality in patients with a LVEF ≤40%. MRAs were associated with a lower risk of mortality in patients with a LVEF >40%, but not in patients with a LVEF ≤40%. The point estimates for these analyses were similar, hence a greater sample size may have increased power sufficiently to demonstrate a benefit in patients with a LVEF ≤40%. Another possibility is that the benefit derived from MRAs is greater earlier in the disease process, and therefore increased survival benefit occurs in patients with a LVEF >40%. The reduced risk of mortality associated with low-dose beta-blockers in patients with a LVEF ≤40% was maintained when excluding patients with concomitant IHD, suggesting that the benefit is related to treating ATTR-CA rather than treating comorbidities, and this is consistent with previous HF trials that demonstrated that improved outcomes were confined to patients with a reduced ejection fraction.^6,7,12^

It has been well established that patients with ATTR-CA have a similar and possibly greater neurohormonal activation than is observed in patients with HF of different aetiologies. Furthermore, elevated neurohormone levels (specifically NT-proBNP and aldosterone) have been associated with a worse prognosis.^23^ It is therefore plausible that patients with ATTR-CA would derive prognostic benefit from neurohormonal modulation. However, a recent position statement by the ESC on the treatment of ATTR-CA recommended the withdrawal of beta-blockers, avoiding ACEi/ARBs, and did not discuss the use of MRAs in patients with ATTR-CA, reflecting the perceived poor tolerability of these agents and lack of trial evidence to support their use (and lack of differentiation between AL and ATTR-CA, the former having greater intolerance).^18^ Several small observational studies have contributed to these recommendations. However, differences in methodology and patient selection could explain our contrasting results. Previous studies have not matched patients, and therefore the worse outcomes in patients treated with HF medications were confounded by disease severity. Our study excluded patients with concomitant polyneuropathy, who often have autonomic disease and hypotension, resulting in a poor tolerance of HF medications.^16,17^ Importantly, our results are supported by a retrospective analysis of the TOPCAT trial, whereby an enriched cohort with echocardiographic characteristics of CA derived benefit from MRA therapy.^22^ This analysis is featured in a recent American College of Cardiology (ACC) consensus document that recommends MRA therapy alongside loop diuretics to augment diuresis.^24^ Our study is the first to sub-categorize ATTR-CA patients by LVEF. The majority of HF patients with a LVEF ≤40% experience chronic adrenergic overstimulation, and higher serum noradrenaline levels than their counterparts with preserved systolic function. A similar pathophysiological mechanism may exist in ATTR-CA, and therefore patients with a LVEF ≤40% could derive benefit from beta-blockade.^23^ Lastly, the majority were treated with bisoprolol (a cardio-selective beta-blocker), which potentially has a different haemodynamic profile to beta-blockers used in previous studies, while still providing neurohormonal modulation, and therefore, the observed benefit could potentially be confined to cardio-selective beta-blockers.

While the observational analyses reported here have limitations in their ability to provide causal estimates of treatments in individuals with ATTR-CA, they do raise the question as to whether there could be benefit from some neurohumoral therapies in such patients and support testing this hypothesis in prospective randomized controlled trials.^22^ While clinical trials are clearly needed, we believe that the data presented in this study call into question the consensus recommendations to discontinue beta-blockers and that neglect to mention MRAs.^18^

## Limitations

There is an unavoidable prescription bias, with comorbid patients with more advanced cardiac disease being treated with more HF medications; but it is also possible that clinicians may have avoided using HF medications in some higher risk patients. Treatment decisions were made on a case-by-case basis, and therefore clinical decisions must factor in each individual’s tolerance of HF medications. It is possible that patients may have discontinued HF medications prior to their first NAC assessment, and this could not be factored into the analysis. Although we performed multivariable adjustment and PS matching to account for confounders known to impact mortality in ATTR-CA, we cannot exclude the possibility of residual confounding. The present study should be considered hypothesis-generating and highlights the urgent need for randomized controlled trials. Some of the estimated HRs generated following pre-specified subgroup analysis were imprecise, and is likely to reflect the unavoidably small sample size. Lastly, a small minority were treated with angiotensin receptor-neprilysin inhibitors or sodium-glucose cotransporter 2 inhibitors and had a short duration of follow-up. Therefore, they were not included in the present study, and further studies will be required to assess these medications in patients with ATTR-CA.

## Conclusions

In summary, in this large cohort of patients with ATTR-CA, a relatively low proportion were treated with conventional HF medications, and those that had a more severe cardiac phenotype were more commonly treated with HF medications. Beta-blockers and ACEi/ARBs were often prescribed at a low dose, and frequently discontinued; in contrast to MRAs which were rarely discontinued. Both regression and PS-matched analyses demonstrated that treatment with a MRA was independently associated with a lower risk of mortality in the overall ATTR-CA population; and PS-matched analysis demonstrated that treatment with a low-dose beta-blocker was independently associated with a lower risk of mortality in patients with a LVEF ≤40%, but these findings require confirmation in prospective randomized controlled trials.

## Supplementary data

Supplementary data are available at *European Heart Journal* online.

## Declarations

### Disclosure of Interest

All authors declare no conflict of interest for this contribution.

## Supplementary Material

ehad347_Supplementary_Data

## Data Availability

The data underlying this article cannot be shared due to restrictions from the institutional ethics committee to protect patient privacy.

## Pre-registered Clinical Trial Number

None supplied.
