# Correction to: Conventional heart failure therapy in cardiac ATTR amyloidosis

**DOI:** 10.1093/eurheartj/ehae140

**Published:** 2024-03-01

This is a correction to: Adam Ioannou, Paolo Massa, Rishi K Patel, Yousuf Razvi, Aldostefano Porcari, Muhammad U Rauf, Anita Jiang, Giacomo Cabras, Stefano Filisetti, Roos E Bolhuis, Francesco Bandera, Lucia Venneri, Ana Martinez-Naharro, Steven Law, Tushar Kotecha, Ruta Virsinskaite, Daniel S Knight, Michele Emdin, Aviva Petrie, Helen Lachmann, Ashutosh Wechelakar, Mark Petrie, Alun Hughes, Nick Freemantle, Philip N Hawkins, Carol Whelan, John J V McMurray, Julian D Gillmore, Marianna Fontana, Conventional heart failure therapy in cardiac ATTR amyloidosis, *European Heart Journal*, Volume 44, Issue 31, 14 August 2023, Pages 2893–2907, https://doi.org/10.1093/eurheartj/ehad347

The following change has been made to the original publication of this manuscript. The **Data Availability** statement has been changed to read: “The data underlying this article cannot be shared due to restrictions from the institutional ethics committee to protect patient privacy.” instead of: “No data were generated or analysed for this manuscript.”

