# Supplementary material for: Conventional heart failure therapy in cardiac ATTR amyloidosis
Source: Eur Heart J. 2023 May 22;44(31):2893–907. doi: 10.1093/eurheartj/ehad347 (PMC10424879; doi:10.1093/eurheartj/ehad347)
Supplement: ehad347_Supplementary_Data [file ehad347_supplementary_data.docx]

**Appendix 1**

**Beta-blockers**

*Reasons for discontinuation:* Of the 285 patients who discontinued treatment with beta-blockers during their follow-up, the reason for discontinuation was documented clearly in 54 cases. The most common reason for discontinuation was bradycardia (n=34), followed by hypotension (n=11). Less common reasons for discontinuation were syncope (n=4), fatigue (n=3), Raynaud’s phenomenon (n=1) and erectile dysfunction (n=1).

*Subgroup analysis of patients with NYHA class III-IV:* Considering the observed benefit in patients with a LVEF ≤40%, further analysis was carried out to assess whether the associated benefit of beta-blockade was also present in patients with more severe HF symptoms. A fourth propensity score-matched cohort was constructed to assess the association between treatment with beta-blockers and risk of mortality in patients with NYHA class III-IV symptoms. This comprised 240 patients (120 treated with beta-blockers vs 120 not treated with beta-blockers), and did not provide convincing evidence for a difference in the risk of mortality between the 2 groups (HR=0.90, 95%CI[0.65-1.26], P=0.551) (Supplementary Table S16).

*Association between dose and survival:* To assess whether there was a dose effect for beta-blocker treatment, patients treated with beta-blockers were divided into those treated with ‘high-dose’ beta-blockers and those treated with ‘low-dose’ beta-blockers, based on the median percentage of the target dose, which was 25%. Therefore ‘high-dose’ was defined as >25% of the target dose, and ‘low-dose’ as ≤25% of the target dose. A fifth propensity score matched cohort was constructed to assess the association between beta-blocker dose and survival. This comprised 676 patients (338 treated with ‘high-dose’ beta-blockers vs 338 treated with ‘low-dose’ beta-blockers) and did not provide convincing evidence for a difference in the risk of mortality between the 2 groups (HR=0.91, 95%CI[0.71-1.16], P=0.448) (Supplementary Table S17).

*Association between change in dosage and survival:* A sixth propensity score matched cohort was constructed to assess the association between a change in the beta-blocker dose and survival. This comprised 682 patients (341 who’s beta-blocker dose was continued/increased vs 341 who’s beta-blocker dose was reduced or their beta-blocker was discontinued) and did not provide convincing evidence for a difference in the risk of mortality between the 2 groups (HR=1.18, 95%CI[0.93-1.50], P=0.162) (Supplementary Table S18).

**Renin-angiotensin system blockers**

*Reasons for discontinuation:* Of the 448 patients who discontinued treatment with ACEi/ARBs during their follow up, the reason for discontinuation was documented clearly in 88 cases. The most common reason for discontinuation was hypotension (n=55) followed by worsening renal function (n=23). Less common reasons for discontinuation were hyperkalaemia (n=4), syncope (n=4) and cough (n=2) .

*Association between dose and survival:* To assess whether there was a dose effect for ACEi/ARB treatment, patients treated with ACEi/ARBs were divided into those treated with ‘high-dose’ ACEi/ARBs and those treated with ‘low-dose’ ACEi/ARBs, based on the median percentage of the target dose, which was 25%. Therefore ‘high-dose’ was defined as >25% of the target dose, and ‘low-dose’ as ≤25% of the target dose. A fourth propensity score matched cohort was constructed to assess the association between ACEi/ARB dose and survival. This comprised 880 patients (440 treated with ‘high-dose’ ACEi/ARB vs 440 treated with ‘low-dose’ ACEi/ARB) and did not provide convincing evidence for a difference in the risk of mortality between the 2 groups (HR=1.17, 95%CI[0.94-1.44], P=0.156) (Supplementary Table S19).

**Combination therapy**

In the overall population, a PS-matched cohort was constructed to compare the association with risk of mortality, between treatment with all 3 classes of HF medications (beta-blockers, ACEi/ARBs and MRAs) and treatment with 2 classes of HF medications (beta-blockers and MRAs). This comprised 360 patients (180 treated with all 3 HF medications vs 180 treated with beta-blockers and MRAs) and did not provide convincing evidence for a difference in the risk of mortality between the 2 groups (HR=0.99, 95%CI[0.70-1.41], P=0.963) (Supplementary Table S20).

**
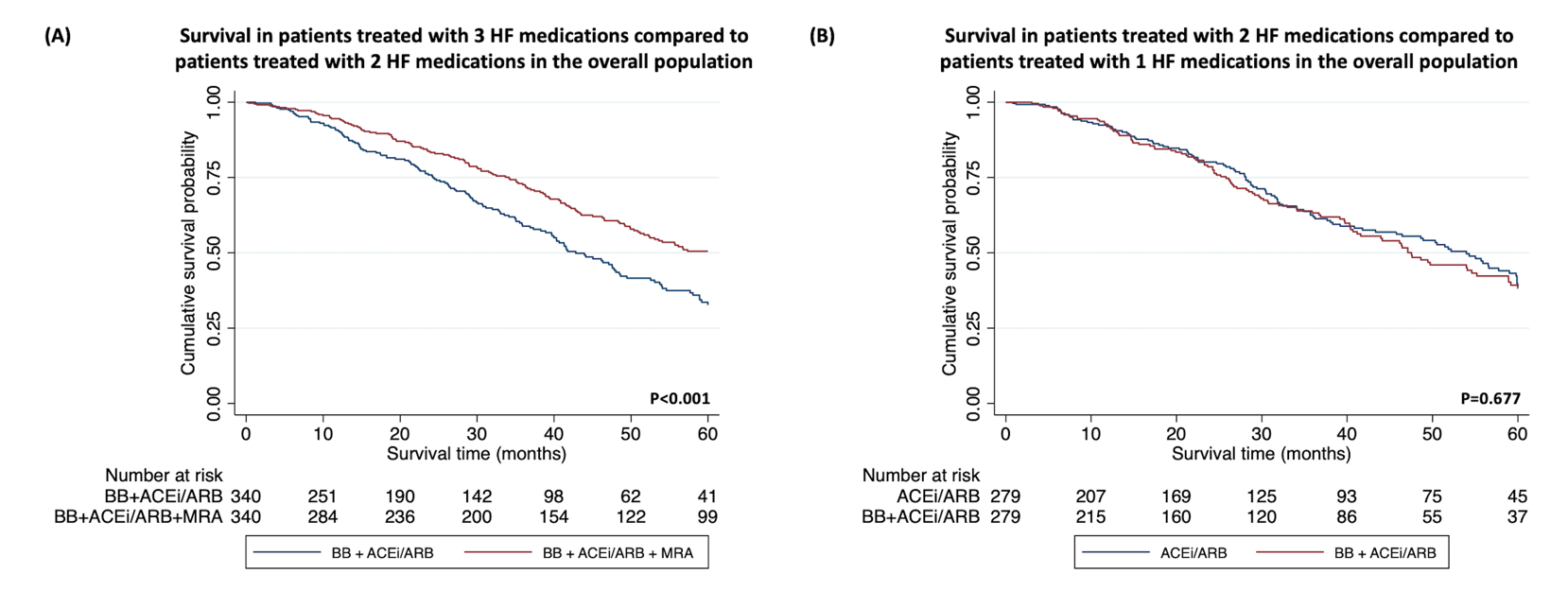
Supplementary Figure S1**

**Supplementary figure S1:** (A) Kaplan-Meier curve comparing survival in patients treated with 3 HF medications (Beta-blockers, ACEi/ARBs and MRAs) to patients treated with 2 HF medications (Beta-blockers and ACEi/ARBs). (B) Kaplan-Meier curve comparing survival in patients treated with 2 HF medications (Beta-blockers and ACEi/ARBs) to patients treated with 1 HF medication (ACEi/ARB).

| **Baseline Characteristics** | **Patients not enrolled into clinical trials or prescribed disease-modifying therapy (n=1904)** | **Patients enrolled into clinical trials and prescribed disease modifying therapy (n=467)** | **P-value** |
| --- | --- | --- | --- |
| Age | 78.0±7.3 | 75.3±6.7 | <0.001 |
| Sex (male) | 1670 (87.7%) | 440 (94.2%) | <0.001 |
| wtATTR | 1439 (75.6%) | 401 (85.9%) | <0.001 |
| hATTR | 465 (24.4%) | 66 (14.1%) | <0.001 |
| AF/flutter | 986 (50.7%) | 237 (48.2%) | 0.688 |
| IHD | 392 (20.6%) | 84 (18.0%) | 0.209 |
| Diabetes mellitus | 304 (16.0%) | 70 (15.0%) | 0.604 |
| Hypertension | 671 (35.2%) | 157 (33.6%) | 0.510 |
| Stroke/TIA | 220 (11.6%) | 38 (8.1%) | 0.034 |
| CKD stage 3-5 | 1076 (56.5%) | 212 (45.4%) | <0.001 |
| **Heart failure severity** |  |  |  |
| NYHA class |  |  | <0.001 |
| 1 | 232 (12.2%) | 85 (18.2%) |  |
| 2 | 1102 (57.9%) | 285 (61.0%) |  |
| 3 | 389 (20.4%) | 46 (9.9%) |  |
| 4 | 30 (1.6%) | 0 (0.0%) |  |
| Missing | 151 | 51 |  |
| NAC stage |  |  | <0.001 |
| 1 | 789 (41.4%) | 297 (63.6%) |  |
| 2 | 723 (38.0%) | 130 (27.8%) |  |
| 3 | 362 (19.0%) | 33 (7.1%) |  |
| Missing | 30 | 7 |  |
| NT-pro-BNP (ng/L) | 3223 (1701-5776) | 2107 (1159-3481) | <0.001 |
| eGFR (ml/min/1.73m²) | 57 (45-70) | 63 (53-76) | <0.001 |
| 6-minute walk test (meters) | 326 (226-411) | 412 (327-467) | <0.001 |
| 6-minute walk test (% predicted) | 67.4±26.9 | 81.6±22.4 | <0.001 |
| Systolic blood pressure (mmHg) | 124.7±22.1 | 126.6±18.7 | 0.50 |
| Diastolic blood pressure (mmHg) | 73.9±12.0 | 76.2±11.5 | <0.001 |
| Heart rate (bpm) | 72.6±13.8 | 70.7±13.0 | 0.015 |
| **Echocardiographic parameters** |  |  |  |
| IVSd (mm) | 16.9±2.5 | 16.9±2.3 | 0.823 |
| PWTd (mm) | 16.4±2.6 | 16.3±2.4 | 0.458 |
| MWT (mm) | 17.1±2.4 | 17.1±2.4 | 0.832 |
| Left atrial area (cm^2^) | 26.4±5.53 | 25.7±5.6 | 0.010 |
| Right atrial area (cm^2^) | 24.5±6.4 | 24.0±6.6 | 0.084 |
| Stroke volume (ml) | 35.7±13.1 | 43.9±15.2 | <0.001 |
| LVEF (%) | 47.7±10.7 | 50.3±9.9 | <0.001 |
| Longitudinal strain (%) | -10.6±3.6 | -11.4±3.7 | <0.001 |
| TAPSE (mm) | 14.7±4.6 | 16.7±5.7 | <0.001 |
| E/e’ | 17.1±6.5 | 15.7±6.0 | <0.001 |
| **Medications** |  |  |  |
| Beta-blockers | 1056 (55.5%) | 257 (55.0%) | 0.867 |
| ACEi/ARBs | 1107 (58.1%) | 255 (54.6%) | 0.166 |
| MRAs | 755 (37.5%) | 170 (36.4%) | 0.197 |

Supplementary Table S1. Baseline characteristics and echocardiographic parameters for patients prescribed disease-modifying therapy or enrolled into clinical trials compared to those not prescribed disease-modifying therapy and not enrolled into clinical trials.

AF = Atrial fibrillation; IHD = Ischaemic heart disease; CKD = Chronic kidney disease; TIA = Transient ischaemic attack; NYHA = New York Heart Association; NAC = National Amyloidosis Centre; NT-pro-BNP = N-terminal pro B-type natriuretic peptide; eGFR = Estimated glomerular filtration rate; IVSd = Interventricular septum in diastole; PWTd = Posterior wall thickness in diastole; MWT = Maximal wall thickness; LVEF = Left ventricular ejection fraction; ACEi = Angiotensin converter enzyme inhibitor; ARB = Angiotensin II receptor blocker; MRA = Mineralocorticoid receptor antagonist.

|  | **Patients treated with beta-blockers (n=1313)** | |  | **Patients treated with ACEi/ARB** | |  |
| --- | --- | --- | --- | --- | --- | --- |
| **Baseline Characteristics** | **Patients who continued beta-blocker treatment**  **(n=1028)** | **Patients who discontinued beta-blocker treatment**  **(n=285)** | **P-value** | **Patients who continued ACEi/ARB treatment**  **(n=914)** | **Patients who continued ACEi/ARB treatment**  **(n=448)** | **P-value** |
| Age | 77.6±6.8 | 76.5±7.2 | 0.006 | 78.1±6.6 | 76.0±6.7 | <0.001 |
| Sex (male) | 909 (88.4%) | 263 (92.3%) | 0.063 | 823 (90.0%) | 401 (89.5%) | 0.759 |
| wtATTR | 811 (78.9%) | 210 (73.7%) | 0.061 | 735 (80.4%) | 332 (74.1%) | 0.008 |
| hATTR | 217 (21.1%) | 75 (26.3%) | 0.061 | 179 (19.6%) | 116 (25.9%) | 0.008 |
| AF/flutter | 599 (58.3%) | 156 (54.7%) | 0.286 | 466 (51.0%) | 262 (58.5%) | 0.009 |
| IHD | 231 (22.5%) | 69 (24.2%) | 0.536 | 208 (22.8%) | 93 (20.8%) | 0.404 |
| Diabetes mellitus | 190 (18.5%) | 51 (17.9%) | 0.821 | 171 (18.7%) | 78 (17.4%) | 0.560 |
| Hypertension | 385 (37.5%) | 94 (33.0%) | 0.166 | 382 (41.8%) | 167 (37.3%) | 0.110 |
| Stroke/TIA | 111 (10.8%) | 26 (9.1%) | 0.413 | 93 (10.2%) | 50 (11.2%) | 0.577 |
| CKD stage 3-5 | 622 (60.5%) | 175 (61.4%) | 0.784 | 507 (55.5%) | 267 (59.6%) | 0.148 |
| **Heart failure severity** |  |  |  |  |  |  |
| NYHA class |  |  | 0.404 |  |  | 0.176 |
| 1 | 112 | 23 |  | 104* | 39 |  |
| 2 | 576 | 179 |  | 511 | 284 |  |
| 3 | 220 | 66 |  | 176 | 99 |  |
| 4 | 14 | 4 |  | 11 | 9 |  |
| Missing |  |  |  |  |  |  |
| NAC stage |  |  | 0.557 |  |  | 0.087 |
| 1 | 408 | 116 |  | 416 | 191 |  |
| 2 | 404 | 118 |  | 328 | 191 |  |
| 3 | 205 | 49 |  | 152 | 65 |  |
| Missing |  |  |  |  |  |  |
| NT-pro-BNP (ng/L) | 3378 (1886-5928) | 3341 (1844-5743) | 0.202 | 2948 (1522-5307) | 3159 (1691-5263) | 0.185 |
| eGFR (ml/min/1.73m²) | 55 (44-69) | 57 (46-68) | 0.664 | 58 (46-70) | 57 (47-69) | 0.533 |
| 6-minute walk test (meters) | 340 (230-427) | 344 (230-414) | 0.503 | 345 (246-430) | 358 (232-427) | 0.954 |
| 6-minute walk test (% predicted) | 68.63±27.1 | 66.2±25.1 | 0.157 | 72.6±26.1 | 69.3±26.8 | 0.058 |
| Systolic blood pressure (mmHg) | 124.3±20.6 | 121.4±18.3 | 0.022 | 126.4±21.3 | 121.7±19.4 | <0.001 |
| Diastolic blood pressure (mmHg) | 74.6±12.9 | 71.9±10.6 | 0.001 | 74.9±12.7 | 72.2±11.3 | <0.001 |
| Heart rate (bpm) | 72.0±14.1 | 68.7±13.2 | <0.001 | 71.9±13.8 | 71.9±13.0 | 0.494 |
| **Echocardiographic parameters** |  |  |  |  |  |  |
| IVSd (mm) | 17.0±2.4 | 16.8±2.4 | 0.170 | 17.0±2.6 | 17.1±2.2 | 0.287 |
| PWTd (mm) | 16.4±2.5 | 16.2±2.4 | 0.129 | 16.3±2.6 | 16.5±2.4 | 0.055 |
| MWT (mm) | 17.2±2.4 | 17.0±2.4 | 0.202 | 17.2±2.5 | 17.2±2.3 | 0.294 |
| Left atrial area (cm^2^) | 26.8±5.5 | 26.1±5.1 | 0.053 | 26.4±5.8 | 26.7±5.3 | 0.239 |
| Right atrial area (cm^2^) | 251±6.4 | 25.2±6.1 | 0.468 | 24.8±6.9 | 25.1±6.0 | 0.224 |
| Stroke volume (ml) | 36.3±14.0 | 37.6±13.3 | 0.118 | 38.5±15.5 | 36.6±12.8 | 0.022 |
| LVEF (%) | 46.8±10.7 | 48.1±10.6 | 0.039 | 47.8±10.7 | 47.6±10.4 | 0.388 |
| Longitudinal strain (%) | -10.5±3.4 | -10.7±3.6 | 0.179 | -10.6±3.5 | -10.8±3.6 | 0.155 |
| TAPSE (mm) | 14.6±4.8 | 15.2±4.7 | 0.038 | 14.9±4.7 | 15.0±4.6 | 0.410 |
| E/e’ | 16.8±6.5 | 16.5±5.8 | 0.215 | 17.0±6.3 | 16.6±6.1 | 0.153 |

Supplementary Table S2. Baseline characteristics and echocardiographic parameters for patients who continued treatment with beta-blockers and ACEi/ARBs compared to those who discontinued treatment with beta-blockers and ACEi/ARBs.

AF = Atrial fibrillation; IHD = Ischaemic heart disease; CKD = Chronic kidney disease; TIA = Transient ischaemic attack; NYHA = New York Heart Association; NAC = National Amyloidosis Centre; NT-pro-BNP = N-terminal pro B-type natriuretic peptide; eGFR = Estimated glomerular filtration rate; IVSd = Interventricular septum in diastole; PWTd = Posterior wall thickness in diastole; MWT = Maximal wall thickness; LVEF = Left ventricular ejection fraction; ACEi = Angiotensin converter enzyme inhibitor; ARB = Angiotensin II receptor blocker.

| **Variables in model** | **Missing data** | **HR (95% CI)** | **P-value** |
| --- | --- | --- | --- |
| Age (years) | 0 | 1.03 (1.01-1.04) | <0.001 |
| Male Sex | 0 | 1.15 (0.93-1.41) | 0.205 |
| hATTR-CA | 0 | 1.82 (1.54-2.14) | <0.001 |
| Ischaemic heart disease | 0 | 1.13 (0.96-1.33) | 0.156 |
| Diabetes mellitus | 0 | 1.11 (0.78-1.04) | 0.268 |
| Hypertension | 0 | 0.90 (0.78-1.04) | 0.166 |
| Atrial fibrillation | 0 | 1.00 (0.87-1.15) | 0.954 |
| NAC stage | 37 |  |  |
| 1 |  | Reference |  |
| 2 |  | 1.78 (1.51-2.09) | <0.001 |
| 3 |  | 2.59 (2.14-3.14) | <0.001 |
| IVSd (mm) | 115 | 1.03 (1.00-1.06) | 0.051 |
| Longitudinal strain (%) | 296 | 1.02 (1.01-1.04) | 0.007 |
| Beta-blocker | 0 | 0.90 (0.78-1.03) | 0.125 |
| ACEi/ARB | 0 | 1.07 (0.93-1.23) | 0.317 |
| MRA | 0 | 0.82 (0.71-0.94) | 0.004 |

Supplementary Table S3. Multivariable cox regression model.

| **Variable** | **Beta-blocker (n = 878)** | **No beta-blocker (n = 878)** | **Standardised difference** |
| --- | --- | --- | --- |
| Age (years) | 77.8 | 77.7 | 0.012 |
| Male sex (%) | 90 | 90 | 0.007 |
| hATTR (%) | 21 | 22 | -0.011 |
| IHD (%) | 19 | 19 | -0.020 |
| DM (%) | 14 | 15 | -0.026 |
| HTN (%) | 34 | 35 | -0.026 |
| AF (%) | 51 | 51 | -0.011 |
| NAC stage 1 (%) | 47 | 45 | 0.053 |
| NAC stage 2 (%) | 38 | 39 | -0.028 |
| NAC stage 3 (%) | 15 | 17 | -0.034 |
| IVSd (mm) | 17.0 | 17.0 | 0.000 |
| LS (mean %) | -11.9 | -11.9 | 0.002 |
| ACEi/ARB (%) | 51 | 54 | -0.066 |
| MRA (%) | 35 | 37 | -0.036 |

Supplementary Table S4. Baseline characteristics of the propensity score matched cohort used to assess the association between treatment with beta-blockers and risk of mortality by comparing patients treated with beta-blockers to those not treated with beta-blockers in the overall population. Continuous variables are expressed as a mean and categorical variable as a percentage.

| **Variable** | **Beta-blocker (n = 169)** | **No beta-blocker (n = 169)** | **Standardised difference** |
| --- | --- | --- | --- |
| Age (years) | 77.2 | 77.1 | 0.014 |
| Male sex (%) | 88 | 88 | 0.018 |
| hATTR (%) | 36 | 35 | 0.025 |
| IHD (%) | 14 | 17 | -0.082 |
| DM (%) | 11 | 15 | -0.087 |
| HTN (%) | 32 | 36 | -0.087 |
| AF (%) | 53 | 51 | 0.024 |
| NAC stage 1 (%) | 31 | 30 | 0.039 |
| NAC stage 2 (%) | 50 | 50 | 0.036 |
| NAC stage 3 (%) | 19 | 23 | -0.088 |
| IVSd (mm) | 17.1 | 17.1 | -0.005 |
| LS (mean %) | -8.5 | -8.8 | 0.067 |
| ACEi/ARB (%) | 48 | 51 | -0.059 |
| MRA (%) | 42 | 43 | -0.024 |

| **Variable** | **Beta-blocker (n = 689)** | **No beta-blocker (n = 689)** | **Standardised difference** |
| --- | --- | --- | --- |
| Age (years) | 77.8 | 77.7 | 0.014 |
| Male sex (%) | 89 | 89 | -0.023 |
| hATTR (%) | 19 | 18 | 0.026 |
| IHD (%) | 20 | 21 | -0.039 |
| DM (%) | 13 | 14 | -0.029 |
| HTN (%) | 34 | 35 | -0.021 |
| AF (%) | 50 | 53 | -0.073 |
| NAC stage 1 (%) | 51 | 50 | 0.046 |
| NAC stage 2 (%) | 35 | 37 | -0.042 |
| NAC stage 3 (%) | 14 | 15 | -0.008 |
| IVSd (mm) | 16.9 | 17.0 | -0.012 |
| LS (mean %) | -12.7 | -12.6 | -0.012 |
| ACEi/ARB (%) | 54 | 55 | -0.017 |
| MRA (%) | 35 | 37 | -0.039 |

Supplementary Table S5. Baseline characteristics of the propensity score matched cohort used to assess the association between treatment with beta-blockers and risk of mortality by comparing patients treated with beta-blockers to those not treated with beta-blockers in patients with a left ventricular ejection fraction **≤**40%. Continuous variables are expressed as a mean and categorical variable as a percentage.

Supplementary Table S6. Baseline characteristics of the propensity score matched cohort used to assess the association between treatment with beta-blockers and risk of mortality by comparing patients treated with beta-blockers to those not treated with beta-blockers in patients with a left ventricular ejection fraction **>**40%. Continuous variables are expressed as a mean and categorical variable as a percentage.

| **Variable** | **ACEi/ARB (n = 891)** | **No ACEi/ARB (n = 891)** | **Standardised difference** |
| --- | --- | --- | --- |
| Age (years) | 77.9 | 77.8 | 0.022 |
| Male sex (%) | 89 | 89 | -0.000 |
| hATTR (%) | 22 | 22 | -0.005 |
| IHD (%) | 19 | 20 | -0.011 |
| DM (%) | 13 | 14 | -0.032 |
| HTN (%) | 31 | 33 | -0.041 |
| AF (%) | 51 | 53 | -0.034 |
| NAC stage 1 (%) | 46 | 44 | 0.038 |
| NAC stage 2 (%) | 37 | 38 | -0.023 |
| NAC stage 3 (%) | 17 | 18 | -0.021 |
| IVSd (mm) | 16.8 | 17.0 | -0.061 |
| LS (mean %) | -12.1 | -11.8 | -0.073 |
| Beta-blocker (%) | 49 | 51 | -0.047 |
| MRA (%) | 34 | 38 | -0.075 |

| **Variable** | **ACEi/ARB (n = 184)** | **No ACEi/ARB (n = 184)** | **Standardised difference** |
| --- | --- | --- | --- |
| Age (years) | 77.8 | 77.4 | 0.062 |
| Male sex (%) | 88 | 87 | 0.016 |
| hATTR (%) | 32 | 33 | -0.035 |
| IHD (%) | 21 | 18 | 0.069 |
| DM (%) | 15 | 18 | -0.073 |
| HTN (%) | 30 | 35 | -0.093 |
| AF (%) | 55 | 54 | 0.022 |
| NAC stage 1 (%) | 27 | 25 | 0.037 |
| NAC stage 2 (%) | 47 | 50 | -0.065 |
| NAC stage 3 (%) | 27 | 25 | 0.037 |
| IVSd (mm) | 17.0 | 17.0 | 0.000 |
| LS (mean %) | -9.1 | -9.2 | 0.014 |
| Beta-blocker (%) | 57 | 58 | -0.022 |
| MRA (%) | 38 | 42 | -0.089 |

Supplementary Table S7. Baseline characteristics of the propensity score matched cohort used to assess the association between treatment with ACEi/ARBs and risk of mortality by comparing patients treated with ACEi/ARBs to those not treated with ACEi/ARBs in the overall population. Continuous variables are expressed as a mean and categorical variable as a percentage.

Supplementary Table S8. Baseline characteristics of the propensity score matched cohort used to assess the association between treatment with ACEi/ARBs and risk of mortality by comparing patients treated with ACEi/ARBs to those not treated with ACEi/ARBs in patients with a left ventricular ejection fraction **≤**40%. Continuous variables are expressed as a mean and categorical variable as a percentage.

| **Variable** | **ACEi/ARB (n = 695)** | **No ACEi/ARB (n = 695)** | **Standardised difference** |
| --- | --- | --- | --- |
| Age (years) | 77.9 | 78.1 | -0.030 |
| Male sex (%) | 88 | 89 | -0.027 |
| hATTR (%) | 19 | 17 | 0.034 |
| IHD (%) | 20 | 20 | -0.022 |
| DM (%) | 13 | 14 | -0.034 |
| HTN (%) | 31 | 33 | -0.043 |
| AF (%) | 52 | 52 | -0.009 |
| NAC stage 1 (%) | 49 | 49 | 0.014 |
| NAC stage 2 (%) | 35 | 36 | -0.009 |
| NAC stage 3 (%) | 15 | 16 | -0.008 |
| IVSd (mm) | 16.8 | 17.0 | -0.091 |
| LS (mean %) | -12.9 | -12.7 | -0.046 |
| Beta-blocker (%) | 47 | 49 | -0.040 |
| MRA (%) | 36 | 38 | -0.039 |

Supplementary Table S9. Baseline characteristics of the propensity score matched cohort used to assess the association between treatment with ACEi/ARBs and risk of mortality by comparing patients treated with ACEi/ARBs to those not treated with ACEi/ARBs in patients with a left ventricular ejection fraction **>**40%. Continuous variables are expressed as a mean and categorical variable as a percentage.

| **Variable** | **MRA (n = 894)** | **MRA (n = 894)** | **Standardised difference** |
| --- | --- | --- | --- |
| Age (years) | 77.1 | 77.2 | -0.007 |
| Male sex (%) | 89 | 89 | 0.014 |
| hATTR (%) | 24 | 23 | 0.016 |
| IHD (%) | 22 | 23 | -0.027 |
| DM (%) | 17 | 16 | 0.021 |
| HTN (%) | 35 | 36 | -0.012 |
| AF (%) | 55 | 57 | -0.036 |
| NAC stage 1 (%) | 42 | 40 | 0.057 |
| NAC stage 2 (%) | 40 | 42 | -0.032 |
| NAC stage 3 (%) | 17 | 19 | -0.032 |
| IVSd (mm) | 17.1 | 17.1 | -0.004 |
| LS (mean %) | -11.3 | -11.4 | 0.005 |
| Beta-blocker (%) | 61 | 62 | -0.021 |
| ACEi/ARB (%) | 60 | 59 | 0.011 |

Supplementary Table S10. Baseline characteristics of the propensity score matched cohort used to assess the association between treatment with MRAs and risk of mortality by comparing patients treated with MRAs to those not treated with MRAs in the overall population. Continuous variables are expressed as a mean and categorical variable as a percentage.

| **Variable** | **MRA (n = 208)** | **MRA (n = 208)** | **Standardised difference** |
| --- | --- | --- | --- |
| Age (years) | 76.9 | 76.9 | 0.001 |
| Male sex (%) | 89 | 89 | -0.015 |
| hATTR (%) | 34 | 34 | -0.000 |
| IHD (%) | 20 | 19 | 0.012 |
| DM (%) | 18 | 22 | -0.084 |
| HTN (%) | 36 | 37 | -0.010 |
| AF (%) | 54 | 56 | -0.039 |
| NAC stage 1 (%) | 31 | 30 | 0.010 |
| NAC stage 2 (%) | 45 | 42 | 0.058 |
| NAC stage 3 (%) | 25 | 28 | -0.077 |
| IVSd (mm) | 16.9 | 17.1 | -0.074 |
| LS (mean %) | -8.8 | -8.5 | -0.086 |
| Beta-blocker (%) | 64 | 68 | -0.081 |
| ACEi/ARB (%) | 59 | 60 | -0.020 |

Supplementary Table S11. Baseline characteristics of the propensity score matched cohort used to assess the association between treatment with MRAs and risk of mortality by comparing patients treated with MRAs to those not treated with MRAs in patients with a left ventricular ejection fraction **≤**40%. Continuous variables are expressed as a mean and categorical variable as a percentage.

| **Variable** | **MRA (n = 667)** | **MRA (n = 667)** | **Standardised difference** |
| --- | --- | --- | --- |
| Age (years) | 77.2 | 77.6 | -0.044 |
| Male sex (%) | 89 | 90 | -0.024 |
| hATTR (%) | 21 | 21 | 0.004 |
| IHD (%) | 21 | 22 | -0.029 |
| DM (%) | 16 | 17 | -0.020 |
| HTN (%) | 35 | 35 | 0.003 |
| AF (%) | 55 | 56 | -0.012 |
| NAC stage 1 (%) | 46 | 44 | 0.054 |
| NAC stage 2 (%) | 38 | 40 | -0.025 |
| NAC stage 3 (%) | 15 | 17 | -0.041 |
| IVSd (mm) | 17.1 | 17.2 | -0.034 |
| LS (mean %) | -12.2 | -12.1 | -0.024 |
| Beta-blocker (%) | 60 | 60 | 0.000 |
| ACEi/ARB (%) | 55 | 56 | -0.012 |

Supplementary Table S12. Baseline characteristics of the propensity score matched cohort used to assess the association between treatment with MRAs and risk of mortality by comparing patients treated with MRAs to those not treated with MRAs in patients with a left ventricular ejection fraction **>**40%. Continuous variables are expressed as a mean and categorical variable as a percentage.

| **Variable** | **Beta-blocker, ACEi/ARB and MRA (n = 340)** | **Beta-blocker and ACEi/ARB (n = 340)** | **Standardised difference** |
| --- | --- | --- | --- |
| Age (years) | 77.1 | 76.9 | 0.041 |
| Male sex (%) | 90 | 89 | 0.057 |
| hATTR (%) | 21 | 23 | -0.057 |
| IHD (%) | 26 | 24 | 0.048 |
| DM (%) | 19 | 20 | -0.007 |
| HTN (%) | 40 | 41 | -0.018 |
| AF (%) | 56 | 56 | 0.000 |
| NAC stage 1 (%) | 40 | 40 | 0.006 |
| NAC stage 2 (%) | 42 | 44 | -0.042 |
| NAC stage 3 (%) | 17 | 16 | 0.048 |
| IVSd (mm) | 17.0 | 17.0 | -0.001 |
| LS (mean %) | -11.4 | -11.4 | 0.002 |

Supplementary Table S13. Baseline characteristics of the propensity score matched cohort used to assess the association between treatment with all 3 classes of HF medications and risk of mortality by comparing patients treated with all 3 classes of HF medications (beta-blockers, ACEi/ARBs and MRAs) to those treated with 2 classes of HF medications (beta-blockers and ACEi/ARBs) in the overall population. Continuous variables are expressed as a mean and categorical variable as a percentage.

| **Variable** | **Beta-blocker and ACEi/ARB (n = 279)** | **ACEi/ARB (n = 279)** | **Standardised difference** |
| --- | --- | --- | --- |
| Age (years) | 78.8 | 78.3 | 0.079 |
| Male sex (%) | 89 | 90 | -0.035 |
| hATTR (%) | 16 | 18 | -0.057 |
| IHD (%) | 23 | 22 | 0.043 |
| DM (%) | 15 | 15 | 0.000 |
| HTN (%) | 44 | 42 | 0.029 |
| AF (%) | 16 | 18 | -0.057 |
| NAC stage 1 (%) | 53 | 49 | 0.086 |
| NAC stage 2 (%) | 32 | 36 | -0.083 |
| NAC stage 3 (%) | 15 | 15 | -0.010 |
| IVSd (mm) | 17.0 | 17.0 | 0.007 |
| LS (mean %) | -12.6 | -12.2 | -0.092 |

Supplementary Table S14. Baseline characteristics of the propensity score matched cohort used to assess the association between treatment with 2 classes of HF medications and risk of mortality by comparing patients treated with all 2 classes of HF medications (beta-blockers and ACEi/ARBs) to those treated with ACEi/ARBs in the overall population. Continuous variables are expressed as a mean and categorical variable as a percentage.

|  | **Deaths in the treated cohort** | **Deaths in the non-treated cohort** |
| --- | --- | --- |
| Beta-blockers in the overall PS-matched population | 308/878 (35.1%) | 343/878 (39.1%) |
| Beta-blockers in the PS-matched population with a LVEF ≤40% | 69/169 (40.8%) | 100/169 (59.2%) |
| Beta-blockers in the PS-matched population with a LVEF >40% | 232/689 (33.7%) | 240/689 (34.8%) |
| ACEi/ARBs in the overall PS-matched population | 355/891 (39.8%) | 299/891 (33.6%) |
| ACEi/ARBs in the PS-matched population with a LVEF ≤40% | 105/184 (57.1%) | 89/184 (48.4%) |
| ACEi/ARBs in the PS-matched population with a LVEF >40% | 260/695 (37.4%) | 215/695 (31.0%) |
| MRAs in the overall PS-matched population | 340/894 (38.0%) | 353 (39.5%) |
| MRAs in the PS-matched population with a LVEF ≤40% | 94/208 (45.2%) | 98/208 (47.1%) |
| MRAs in the PS-matched population with a LVEF >40% | 236/667 (35.3%) | 246/667 (36.9%) |
|  | **Deaths in the cohort treated with 3 HF medications** | **Deaths in the cohort treated with beta-blockers and ACEi/ARB** |
| 3 HF medications vs 2 HF mediations in the overall population | 123/340 (36.2%) | 139/340 (40.9% |
|  | **Deaths in the cohort treated with beta-blockers and ACEi/ARB** | **Deaths in the cohort treated with ACEi/ARB** |
| 2 HF medications vs 1 HF mediations in the overall population | 103/279 (36.9%) | 106/279 (38.0%) |

Supplementary Table S15. Summary of the number of events and event rates in both the treated and non-treated cohorts within each initial propensity score matched analysis.

LVEF = Left ventricular ejection fraction; ACEi = Angiotensin converter enzyme inhibitor; ARB = Angiotensin II receptor blocker; MRA = Mineralocorticoid receptor antagonist.

| **Variable** | **Beta-blocker (n = 120)** | **No beta-blocker (n = 120)** | **Standardised difference** |
| --- | --- | --- | --- |
| Age (years) | 79.4 | 79.0 | 0.061 |
| Male sex (%) | 86 | 84 | 0.046 |
| hATTR (%) | 34 | 36 | -0.035 |
| IHD (%) | 23 | 21 | 0.060 |
| DM (%) | 16 | 15 | 0.023 |
| HTN (%) | 36 | 34 | 0.035 |
| AF (%) | 51 | 57 | -0.117 |
| NAC stage 1 (%) | 28 | 27 | 0.037 |
| NAC stage 2 (%) | 46 | 43 | 0.050 |
| NAC stage 3 (%) | 26 | 30 | -0.093 |
| IVSd (mm) | 17.5 | 17.4 | 0.040 |
| LS (mean %) | -9.6 | -9.5 | -0.044 |
| ACEi/ARB (%) | 49 | 51 | -0.033 |
| MRA (%) | 41 | 46 | -0.101 |

Supplementary Table S16. Baseline characteristics of the propensity score matched cohort used to assess the association between treatment with beta-blockers and risk of mortality in a subgroup of patients with New York Heart Association (NYHA) class III-IV symptoms by comparing patients treated with beta-blockers to those not treated with beta-blockers. Continuous variables are expressed as a mean and categorical variable as a percentage.

| **Variable** | **High-dose beta-blocker (n = 338)** | **Low-dose beta-blocker (n = 338)** | **Standardised difference** |
| --- | --- | --- | --- |
| Age (years) | 76.9 | 76.7 | 0.035 |
| Male sex (%) | 89 | 89 | 0.010 |
| hATTR (%) | 23 | 24 | -0.028 |
| IHD (%) | 25 | 23 | 0.035 |
| DM (%) | 19 | 20 | -0.022 |
| HTN (%) | 41 | 43 | -0.054 |
| AF (%) | 65 | 64 | 0.019 |
| NAC stage 1 (%) | 36 | 36 | -0.012 |
| NAC stage 2 (%) | 44 | 46 | -0.024 |
| NAC stage 3 (%) | 20 | 18 | 0.045 |
| IVSd (mm) | 16.9 | 16.9 | 0.010 |
| LS (mean %) | -10.4 | -10.5 | 0.020 |
| ACEi/ARB (%) | 66 | 67 | -0.019 |
| MRA (%) | 48 | 49 | -0.006 |

Supplementary Table S17. Baseline characteristics of the propensity score matched cohort used to assess the association between treatment with high-dose and low-dose beta-blockers and risk of mortality by comparing patients treated with high-dose beta-blockers and those treated with low-dose beta-blockers in the overall population. Continuous variables are expressed as a mean and categorical variable as a percentage.

| **Variable** | **Beta-blocker dose continued or increased (n = 341)** | **Beta-blocker dose reduced or discontinued (n = 341)** | **Standardised difference** |
| --- | --- | --- | --- |
| Age (years) | 75.8 | 76.4 | -0.086 |
| Male sex (%) | 92 | 91 | 0.021 |
| hATTR (%) | 30 | 26 | 0.078 |
| IHD (%) | 21 | 21 | 0.000 |
| DM (%) | 20 | 18 | 0.030 |
| HTN (%) | 31 | 33 | -0.050 |
| AF (%) | 60 | 58 | 0.036 |
| NAC stage 1 (%) | 43 | 41 | 0.024 |
| NAC stage 2 (%) | 43 | 42 | 0.024 |
| NAC stage 3 (%) | 14 | 17 | -0.065 |
| IVSd (mm) | 17.0 | 17.0 | -0.022 |
| LS (mean %) | -10.9 | -10.7 | -0.043 |
| ACEi/ARB (%) | 74 | 70 | 0.098 |
| MRA (%) | 60 | 57 | 0.077 |

Supplementary Table S18. Baseline characteristics of the propensity score matched cohort used to assess the association between reducing or discontinuing beta-blocker therapy and the risk of mortality by comparing patients treated with continued or increased beta-blocker therapy and those treated with reduced or discontinued beta-blocker therapy in the population of patients treated with beta-blockers. Continuous variables are expressed as a mean and categorical variable as a percentage.

| **Variable** | **High-dose ACEi/ARBs (n = 440)** | **Low-dose ACEi/ARBs (n = 440)** | **Standardised difference** |
| --- | --- | --- | --- |
| Age (years) | 77.1 | 76.9 | 0.022 |
| Male sex (%) | 92 | 91 | 0.049 |
| hATTR (%) | 20 | 22 | -0.044 |
| IHD (%) | 23 | 22 | 0.005 |
| DM (%) | 19 | 20 | -0.017 |
| HTN (%) | 39 | 41 | -0.032 |
| AF (%) | 55 | 54 | 0.018 |
| NAC stage 1 (%) | 48 | 48 | -0.005 |
| NAC stage 2 (%) | 37 | 38 | -0.005 |
| NAC stage 3 (%) | 15 | 14 | 0.013 |
| IVSd (mm) | 17.1 | 17.0 | 0.023 |
| LS (mean %) | -10.6 | -10.7 | 0.040 |
| Beta-blocker (%) | 64 | 63 | 0.024 |
| MRA (%) | 45 | 44 | 0.027 |

Supplementary Table S19. Baseline characteristics of the propensity score matched cohort used to assess the association between treatment with high-dose and low-dose ACEi/ARBs and risk of mortality by comparing patients treated with high-dose ACEi/ARBs and those treated with low-dose ACEi/ARBs in the overall population. Continuous variables are expressed as a mean and categorical variable as a percentage.

| **Variable** | **Beta-blocker, ACEi/ARB and MRA (n = 180)** | **Beta-blocker and MRA (n = 180)** | **Standardised difference** |
| --- | --- | --- | --- |
| Age (years) | 78.0 | 77.3 | 0.124 |
| Male sex (%) | 89% | 91% | -0.055 |
| hATTR (%) | 24% | 24% | 0.000 |
| IHD (%) | 22% | 22% | -0.013 |
| DM (%) | 12% | 16% | -0.111 |
| HTN (%) | 28% | 32% | -0.073 |
| AF (%) | 63% | 59% | 0.080 |
| NAC stage 1 (%) | 34% | 39% | -0.092 |
| NAC stage 2 (%) | 43% | 41% | 0.056 |
| NAC stage 3 (%) | 22% | 21% | 0.041 |
| IVSd (mm) | 17.0 | 17.1 | -0.020 |
| LS (mean %) | -11.1 | -11.2 | 0.016 |

Supplementary Table S20. Baseline characteristics of the propensity score matched cohort used to assess the association between treatment with all 3 classes of HF medications and risk of mortality by comparing patients treated with all 3 classes of HF medications (beta-blockers, ACEi/ARBs and MRAs) to those treated with 2 classes of HF medications (beta-blockers and MRAs) in the overall population. Continuous variables are expressed as a mean and categorical variable as a percentage.

| **Baseline Characteristics** | **Overall study population (n=2371)** | **Patients treated with ARNI and/or SGLT2i (n=59)** | **P-value** |
| --- | --- | --- | --- |
| Age | 77.5±7.3 | 76.7±6.6 | 0.216 |
| Sex (male) | 2110 (90.0%) | 54 (91.5%) | 0.538 |
| AF/flutter | 1223 (51.6%) | 42 (71.2%) | 0.003 |
| IHD | 476 (20.1%) | 8 (13.6%) | 0.216 |
| Diabetes mellitus | 374 (15.8%) | 17 (28.8%) | 0.007 |
| Hypertension | 828 (34.9%) | 21 (35.6%) | 0.915 |
| Stroke/TIA | 109 (4.6%) | 4 (6.8%) | 0.316 |
| CKD stage 3-5 | 1288 (54.3%) | 40 (67.8%) | 0.040 |
| **Heart failure severity** |  |  |  |
| NYHA class |  |  | 0.876 |
| 1 | 317 (13.4%) | 7 (11.9%) |  |
| 2 | 1387 (58.5%) | 30 (50.8%) |  |
| 3 | 435 (18.3%) | 10 (16.9%) |  |
| 4 | 30 (1.3%) | 0 (0.0%) |  |
| Missing | 202 | 12 |  |
| NAC stage |  |  | 0.396 |
| 1 | 1086 (45.8%) | 28 (47.4%) |  |
| 2 | 853 (36.0%) | 17 (28.8%) |  |
| 3 | 395 (16.7%) | 13 (22.0%) |  |
| Missing | 37 | 1 |  |
| NT-pro-BNP (ng/L) | 2925 (1530-5321) | 2913 (1447-5465) | 0.995 |
| eGFR (ml/min/1.73m²) | 58 (46-71) | 53 (43-66) | 0.077 |
| **Echocardiographic parameters** |  |  |  |
| IVSd (mm) | 16.9±2.4 | 16.6±2.7 | 0.461 |
| PWTd (mm) | 16.3±2.5 | 16.0±2.4 | 0.342 |
| MWT (mm) | 17.1±2.4 | 16.9±2.5 | 0.744 |
| Left atrial area (cm^2^) | 26.2±5.5 | 26.8±4.1 | 0.421 |
| Right atrial area (cm^2^) | 24.5±6.5 | 24.5±6.5 | 0.880 |
| Stroke volume (ml) | 37.3±13.9 | 35.7±14.3 | 0.597 |
| LVEF (%) | 48.2±10.6 | 43.1±9.8 | <0.001 |
| Longitudinal strain (%) | -10.8±3.6 | -9.4±3.0 | 0.007 |
| TAPSE (mm) | 15.1±4.9 | 13.3±3.9 | 0.144 |
| E/e’ | 16.8±6.4 | 16.6±6.2 | 0.857 |

Supplementary Table S21. Baseline characteristics and echocardiographic parameters for patients prescribed angiotensin receptor-neprilysin inhibitors or sodium-glucose co-transporter-2 inhibitors compared to those included in the present study.

AF = Atrial fibrillation; IHD = Ischaemic heart disease; CKD = Chronic kidney disease; TIA = Transient ischaemic attack; NYHA = New York Heart Association; NAC = National Amyloidosis Centre; NT-pro-BNP = N-terminal pro B-type natriuretic peptide; eGFR = Estimated glomerular filtration rate; IVSd = Interventricular septum in diastole; PWTd = Posterior wall thickness in diastole; MWT = Maximal wall thickness; LVEF = Left ventricular ejection fraction.
